# Phylogenetically diverse Mucorales–Mycetohabitans endosymbiotic interactions identified from whole-genome sequencing using a targeted metagenomic assembly pipeline

**DOI:** 10.1099/mgen.0.001746

**Published:** 2026-07-14

**Authors:** Jack B. Gregory, Jamie W. Harrison, Jessie K. Uehling, Rhys A. Farrer, Elizabeth R. Ballou

**Affiliations:** 1 Medical Research Council Centre for Medical Mycology, University of Exeter, Geoffrey Pope Building, Stocker Road, Exeter EX4 4QD, UK; 2 Department of Botany and Plant Pathology, Oregon State University, Corvallis, Oregon, USA

**Keywords:** cophylogeny, host–bacteria interaction, metagenomes, Mucorales, *Mycetohabitans*, symbionts

## Abstract

Endosymbiotic bacteria of the genus *Mycetohabitans* are obligate intracellular associates of Mucorales fungi, yet the understanding of their diversity, distribution and evolutionary dynamics is in its infancy. By screening 1,696 public sequencing datasets from Mucorales fungi, we detected *Mycetohabitans* in 46 fungal accessions spanning 5 host taxa across the fungal genera *Rhizopus* and *Apophysomyces*. These included 13 previously unreported associations. Genome reconstruction yielded 38 *Mycetohabitans* metagenome-assembled genomes (MAGs), of which 34 were of high quality. Incorporating these MAGs into genome-based species delimitation expanded known *Mycetohabitans* diversity from four to nine species-level clusters, including novel host-associated lineages. Re-examination of fungal host identities revealed frequent misidentification of isolates in fungal collection catalogues and/or misannotation in GenBank, with nearly a quarter of positive datasets requiring correction through internal transcribed spacer and genome-scale verification. Host–symbiont associations were non-random under this revised framework, with significant structure detected by contingency analysis and ParaFit. MAG-focused pangenome analysis revealed an open pangenome and mosaic lineage-associated functional traits, including variation in metabolism, secretion, cell-envelope systems, metal resistance, antimicrobial-resistance-associated functions and mobile elements. The most distinctive lineage comprised two *Apophysomyces*-associated MAGs, provisionally named *M. apophysomyceticola*, which showed pronounced genome reduction compared with other sampled *Mycetohabitans* spp. and loss of multiple central metabolic, nutrient assimilation, cofactor biosynthesis, catabolic, stress-response and defence pathways, consistent with reduced metabolic flexibility and increased host dependence. Together, these results show that *Mycetohabitans* symbioses are more geographically widespread, taxonomically diverse and functionally differentiated than previously recognized. More broadly, this work demonstrates the value of public sequencing repositories for uncovering hidden fungal–bacterial symbioses, while emphasizing that repository-derived patterns must be interpreted considering host misidentification, uneven sampling and incomplete metadata. Overall, our work establishes a global framework for *Mycetohabitans* diversity and function, with implications for fungal ecology, evolution and clinical mycology.

**Impact Statement**

Many Mucorales fungi, including species of *Rhizopus* and *Apophysomyces*, harbour intracellular endobacteria, particularly of the *Mycetohabitans* genus, that influence fungal development, virulence and ecological interactions. Research has focused primarily on *Rhizopus microsporus–Mycetohabitans rhizoxinica*, a model association with relevance to plant disease, opportunistic human infection and the evolution of fungal endosymbiosis. Despite their importance, bacterial symbioses within these fungal genera remain poorly characterized in terms of host diversity, endosymbiont clade composition and functional diversity. By mining 1,696 public fungal sequencing datasets for the presence of *Mycetohabitans* genomes, we uncovered previously unreported host–symbiont associations, corrected frequent fungal host misidentifications and reconstructed 38 endosymbiont metagenome-assembled genomes (MAGs). Genome-based species delimitation expanded the known diversity of *Mycetohabitans* from four to nine species-level clusters and revised the interpretation of several host–symbiont associations. Comparative analyses revealed non-random host–symbiont structure, an open MAG-based pangenome and lineage-associated functional variation across the genus. Notably, we identified a distinct *Apophysomyces*-associated lineage, provisionally named *Mycetohabitans apophysomyceticola*, with pronounced genome reduction and extensive loss of metabolic, nutrient assimilation and defence pathways, consistent with increased host dependence. These findings expand the known ecological and evolutionary scope of *Mycetohabitans* endosymbioses and demonstrate the value of public sequencing repositories for discovering hidden fungal–bacterial partnerships. They also highlight the need for genome-based host verification and targeted sampling in environmental and clinical contexts.

## Data Summary

The authors confirm all supporting data, code and protocols have been provided within the article or through supplementary data files. All generated *Mycetohabitans* metagenome-assembled genomes have been deposited in Zenodo (https://doi.org/10.5281/zenodo.18216012). All code scripts used have been deposited in GitHub (https://github.com/Jack-Gregory-Bioinformatics/Endosymbiont_Hunting). All supplementary files have been deposited in the Microbiology Society’s data repository Figshare account: https://doi.org/10.6084/m9.figshare.32106079[1].

## Introduction

Endosymbiotic bacteria are increasingly found in fungi [2,5]. Within the Mucoromycota, reports suggest that 20% of environmental isolates contain endosymbiotic bacteria [6,7]. While new endohyphal bacteria are now being discovered [8,10], historically accounts of endobacteria in Mucoromycota belong largely to one of two classes: the *Burkholderia*-related endobacteria (BRE) and the *Mycoplasma*-related bacteria [6,7, 11,14]. Fungi of the order Mucorales are filamentous fungi associated with soil, decaying vegetation and compost, environments that facilitate bacterial interactions as well as exposure to crops and humans [15], and their bacterial endosymbionts have been shown to modulate fungal behaviour and pathogenesis towards plants and humans [8,16,19].

The most common BRE endosymbionts of Mucorales are *Mycetohabitans* spp. [20,23]. Fungal sporulation allows for vertical transmission of the endosymbiont, and endosymbionts use diverse mechanisms to maintain themselves within their fungal hosts, in part by altering host fitness costs and benefits. For example, BREs can manipulate the fungal reproductive cycle, fully controlling asexual reproduction and partially controlling sexual reproduction, as shown for *Rhizopus microsporus–Mycetohabitans* endosymbioses [24]. Endosymbionts can enhance fungal host survival through the secretion of bioactive molecules that provide protection against natural predators, such as amoeba [8,25]. Functional studies have also revealed defensive traits in *Mycetohabitans* and phylogenetically related *R. microsporus–Ralstonia pickettii* endosymbiosis that confer increased host resistance to predation and antifungal stress [8,25]. The facultative nature of the BREs also suggests that horizontal transfer between isolates of the same species or between host species may be possible.

Members of the Mucorales can cause mucormycosis, a rare but frequently fatal infection (exceeding 50% and reaching nearly 100% in disseminated cases) [26,29]. Mucormycosis has a higher prevalence in immunocompromised individuals, such as organ transplant recipients, patients on immunosuppressive drugs and autoimmune sufferers [30,31]. The global burden of mucormycosis remains elusive due to inconsistent reporting and limited surveillance, yet incidence appears to be rising, especially in regions such as India where rates are estimated at 140 cases per million per year, which is 70 times the global average [32,35]. While *Rhizopus* spp. are the most common agents of mucormycosis, other *Mucorales* genera, including *Lichthemia*, *Apophysomyces* and *Rhizomucor*, are also implicated in community- and hospital-acquired outbreaks [36,37]. We lack a true understanding of the diversity and impact of bacterial endosymbionts in fungal clinical isolates, but recent work has identified *Mycetohabitans* spp. directly in clinical isolates [38] and as a cause of sepsis in mucormycosis patients [39,41]. Additionally, *Mycetohabitans* genes have been identified in Sequence Read Archive (SRA) data from clinical isolates of *Apophysomyces* [42].

Our understanding of the diversity, distribution and functional implications of these endobacteria remains in its infancy, particularly in clinical isolates, with new fungal-endosymbiont relationships being reported as detection methods improve [7,9, 21, 42,46]. Previous studies of *Mycetohabitans*–fungal interactions have provided important insights using a range of approaches, including culturing and sequencing isolated endosymbionts [42,47,50], targeted 16S rRNA amplicon sequencing of host–symbiont DNA [48,51] and FISH microscopy to visualize bacteria within fungal cells [6]. Each of these methods has been valuable for establishing the presence and identity of endosymbionts; culturing enables full genome sequencing of isolated strains, 16S surveys provide broad taxonomic coverage and FISH offers direct confirmation of intracellular localization. At the same time, these approaches have inherent limitations, such as difficulties in recovering unculturable symbionts, restricted resolution beyond the 16S marker or lack of species-level discrimination. Building on this foundation, genome-resolved approaches applied directly to host sequencing data now offer a complementary way to capture endosymbiont diversity and functional potential without the need for prior isolation [52]. However, sequencing and assembling endosymbiont genomes from complex host data is methodologically challenging as bacterial reads are typically embedded within a much larger background of fungal DNA. Accurate recovery therefore requires specialized bioinformatic workflows to disentangle mixed signals, address reduced or atypical genome features and mitigate issues such as fragmented assemblies, missing plasmids and potential contamination.

With the increasing use of whole-genome sequencing in clinical and environmental mycology, large numbers of *Mucorales* sequencing datasets are now publicly available through resources such as the NCBI SRA. These datasets, generated primarily for fungal identification and comparative genomics, represent a valuable resource that may also contain signal from associated bacterial endosymbionts [42,53,56].

Building on this wealth of genomic data, we developed a pipeline for partial to complete endosymbiont genome assembly construction and applied it to such data across 1,696 SRAs covering 25 *Mucorales* species, bypassing the need to isolate and sequence the endosymbionts independently from their hosts, inspired by an approach carried out by Twort *et al*., who screened public Lepidoptera whole genome sequencing (WGS) for evidence of endosymbionts [52]. To validate the pipeline, we specifically searched for evidence of *Mycetohabitans* spp., as they are well-established endosymbionts of *R. microsporus* [57]. Using phylogenetics, we assessed strain diversity and potential clinical relevance. Our results offer new insight into the hidden diversity of *Mycetohabitans* endosymbionts associated with *Mucorales* and highlight the need for their inclusion in fungal pathogenesis studies.

## Methods

### Dataset construction

All samples included in this study were downloaded from the NCBI SRA. To identify samples for screening, a systematic search for raw sequence reads was carried out in the NCBI SRA database, using the following search term ‘(((((((((((mucor) OR rhizopus) OR rhizomucor) OR absidia) OR lichtheimia) OR apophysomyces) OR cunninghamella) OR saksenaea) OR syncephalastrum) OR cokeromyces) OR mucormycosis) OR mucorales’. This search returned all Mucorales datasets present in the NCBI SRA database. The search was limited to raw sequence data from all samples published to the SRA database, of which the final search was performed in May 2025. Retrieved datasets were further restricted to Illumina paired-end data and excluded if they were classified as (i) RNA-seq data, (ii) amplicon data, (iii) bisulphite sequencing or (iv) metagenomic data. This resulted in a total of 1,696 samples for analysis, covering 211 species (File S1, available in the online Supplementary Material). The NRRL August 2025 Fungi Catalog was used to add sample geographic origin to NRRL samples present in the processed SRAs. SRA Toolkit v3.1.0 [58] was used to download the reads from each accession.

### Identification of *Mycetohabitans* reads in public SRA datasets

Reads were quality-checked using FastQC v0.12.1 [59], trimmed and quality-filtered by Trimmomatic v0.39 [60] with parameters (LEADING: 20 TRAILING:20 SLIDINGWINDOW:5 : 20 MINLEN:50). The trimmed paired-end reads then underwent another round of quality checking using FastQC and were assigned taxonomic labels with Kraken2 v2.1.3 [61] and MetaPhlAn v4.1.1 [62]. Kraken2 was run using a confidence threshold of 0.05, a mpa style output and a custom database which contained: (i) the standard kraken database, (ii) RefSeq archaea database, (iii) RefSeq bacterial database, (iv) RefSeq fungi database, (v) RefSeq viral database, (vi) Univec core database, (vii) available clinically relevant Mucorales genomes and (viii) available *Mycetohabitans* genomes. MetaPhlAn was run using the analysis type rel_ab_w_read_stats, which provides the relative abundance and an estimate of read numbers originating from each clade. The resulting outputs were screened for lines matching *Mycetohabitans*. Based on the Kraken2 results, datasets that contained a conservative >1,000 hits to *Mycetohabitans* were taken forward for further analysis.

To assess potential biological contamination, Kraken2 taxonomic assignments were screened for reads classified as Arthropoda or Nematoda, used here as sentinel groups because metazoan carryover, particularly dust mite-like contamination, is a recurrent feature of public fungal sequencing datasets. Broader taxonomic assignment profiles were also reviewed for unexpected non-fungal/non-bacterial read fractions. No *Mycetohabitans*-positive sequencing run contained more than 100 reads assigned to either sentinel group.

### Identification and assembly of *Mycetohabitans* genomic information

SRA experiments that met our screening criteria and had indications for the presence of *Mycetohabitans* based on Kraken2 identification of reads were processed as follows (Fig. S1).

For each SRA run, trimmed paired-end reads were assembled using SPAdes v3.15.5 [63], running in a metagenomic state with the argument ‘--meta’, with k-mer sizes of 21, 33 and 55 used.

To identify assembled contigs originating from *Mycetohabitans*, a reference blast nucleotide database containing previously published genomes of *Mycetohabitans* was constructed. Only *Mycetohabitans* genomes marked as complete in the NCBI RefSeq database were included. A total of nine *Mycetohabitans* genomes, comprising five *Mycetohabitans endofungorum* and four *Mycetohabitans rhizoxinica* genomes, were downloaded from the NCBI (August 2025). A complete list of the genomes included is shown in File S2. Resulting contigs were queried against the custom *Mycetohabitans* blast nucleotide database using blast v2.16 [64,66] with the ‘megablast’ task, an *E*-value threshold of 1e-10, minimum percent identity of 70%, query coverage ≥50% and minimum alignment length of 500 bp. Contigs with significant blast hits were retained as candidate bacterial contigs, while those without were categorized as non-bacterial. Contigs were partitioned accordingly using SeqKit v2.10 [67].

Both bacterial and non-bacterial contig bins were subjected to further taxonomic classification with Kraken2 v2.1.3 at a confidence threshold of 0.3, against the custom database. Assembly quality was assessed using QUAST v5.2.0 [68], while completeness and gene content were evaluated with BUSCO v5.5.0 [69] using the bacterial (odb10) and fungal (odb10) reference lineages. Bacterial bins were assessed with both bacterial and fungal BUSCO datasets to check for potential cross-lineage contamination and vice versa for the non-bacterial bins.

### Genome-based species delimitation of *Mycetohabitans* spp.

Overall genomic relatedness among *Mycetohabitans* strains was assessed using average nucleotide identity (ANI) and digital DNA–DNA hybridization (dDDH) [70,71]. The analysis included the newly generated bacterial metagenome-assembled genomes (MAGs) recovered in this study and additional publicly available *Mycetohabitans* genomes downloaded from public databases; details of the additional downloaded genomes included are provided in (File S3). ANI was calculated using the OrthoANI algorithm in the command-line implementation of the Orthologous Average Nucleotide Identity Tool (OAT) [72], using all-versus-all pairwise comparisons of genome assemblies in FASTA format. dDDH values were obtained using the Genome-to-Genome Distance Calculator v3.0 of the web-based DSMZ service (http://ggdc.dsmz.de) and the recommended Formula 2 [71]. Resulting ANI and dDDH values were collated into pairwise similarity matrices, which were visualized as heatmaps in R. Final figure layouts were prepared in Adobe Illustrator. Species-level relatedness was interpreted using the commonly applied thresholds of 95% ANI and 70% dDDH, with dotted lines used to indicate these thresholds.

### Identification of fungal host

To identify fungal signal within metagenomic assemblies, ITSx v1.1.3 [73] was used to extract and classify internal transcribed spacer (ITS) regions from non-bacterial assigned contigs. Sequences were then blastn against the core_nt database using the NCBI blastn suite [64]. Non-bacterial contigs were reanalysed against the custom Kraken2 database to validate the fungal identifications inferred from ITS blastn. As an additional check, we compared the total base-pair content of each non-bacterial contig bin with the expected genome size of the assigned fungal species.

### Phylogenetic reconstruction

To create a bacterial endosymbiont phylogeny, bacterial MAGs resulting from the bacterial contig binning were processed using the pipeline BUSCO phylogenomics developed by Jamie McGowan (2023) and available at https://github.com/jamiemcg/BUSCO_phylogenomics. In summary, the bacterial MAGs were used to identify single-copy genes with BUSCO v5.5.0 in genome mode, using the bacteria odb10 database. Amino acid sequences of each retained BUSCO gene were aligned independently using muscle v5.1 [74] and trimmed with trimAl v1.4.1 [75] under the ‘automated1’ option. Trimmed alignments were concatenated in order of BUSCO ID to generate a supermatrix of 36,008 amino acid positions, and an accompanying partition file was produced. Maximum-likelihood inference was performed with RAxML-NG v1.2.2 [76] using the command ‘raxml-ng --all --msa SUPERMATRIX.phylip --model partitions.raxml.txt --prefix supermatrix_tree --threads 16 --seed 12345 --bs-trees 1000 --bs-metric fbp,tbe’. Bootstrap support was estimated from 1,000 replicates (both Felsenstein bootstrap proportions and transfer bootstrap expectation). The best-scoring maximum likelihood tree with support values was used for phylogeny visualization. *Candidatus Glomeribacter gigasporarum* strain BEG34 (GCF_000227585.1) was included as an outgroup to root the bacterial phylogeny, with the outgroup branch removed from the final visualization for clarity.

To create a fungal host phylogeny, for each sample, the non-bacterial contig bin was analysed using the same pipeline as above but utilizing the fungi odb10 database for single-copy gene identification. The resulting supermatrix contained 251,850 amino acid positions and a maximum-likelihood tree was produced with the same arguments as for the bacterial tree. *Linnemannia elongata* strain NVP64 (GCA_036320915.1) was included as an outgroup to root the tree, although the outgroup branch was removed from the final visualization for clarity.

The final trees with bootstrap support values were visualized in Interactive Tree of Life (iTOL) [77] and then combined manually to create a bacterial endosymbiont-fungal host tanglegram.

### Host–symbiont association analysis and co-phylogeny

Identified associations between fungal hosts and *Mycetohabitans* endosymbionts were quantified using a curated dataset of paired sample identifiers and taxonomic assignments. Using R (v4.5.1; R Core Team 2021), all observed fungal–bacterial pairings were tabulated to generate a contingency table of host species versus symbiont species. The frequency of each pairing was calculated, and a global test of independence between fungal host identity and bacterial symbiont identity was performed using a chi-square test.

Pairwise association frequencies were visualized as a heatmap using the R package *pheatmap*, with hierarchical clustering applied to both fungal and bacterial dimensions to identify patterns of co-occurrence. Zero-count cells were visually suppressed to enhance interpretability. All plots were generated using the *tidyverse*, *pheatmap* and *grid* packages [78].

To assess congruence between fungal host and bacterial symbiont phylogenies, co-phylogenetic analyses were performed using the ParaFit method implemented in the R package *ape* [79]. Maximum-likelihood phylogenies from the previous step for fungal hosts and bacterial symbionts were used to calculate pairwise patristic distance matrices. Host–symbiont associations were represented as a binary identity matrix linking matched sample identifiers.

ParaFit was run with 9,999 permutations to test for global congruence between host and symbiont phylogenies and to evaluate the significance of individual host–symbiont links. Negative eigenvalues in the distance matrices were corrected using the Cailliez method. For each association, ParaFitLink statistics (F1 and F2) were calculated, and resulting *P*-values were adjusted for multiple testing using the Benjamini–Hochberg false discovery rate (FDR) correction. Associations were classified as supporting co-phylogeny (FDR-adjusted *P*<0.05 and positive F1 statistic), discordant (negative F1 statistic) or not significant. This statistical framework enabled assessment of both global and pairwise patterns of host–symbiont co-diversification.

### Pangenome analysis and functional enrichment

Pangenomes of *Mycetohabitans* MAGs were constructed and visualized using anvi’o v8 [80]. For each MAG FASTA file, contigs databases were generated with anvi-gen-contigs-database, which included anvi’o’s internal gene calling using an extension of pyrodigal [81,82]. Subsequently, we performed a series of annotation steps on each contigs database: anvi-run-hmms to identify single-copy core genes and other hidden Markov model profiles, anvi-run-ncbi-cogs for COG functional annotation [83,84], anvi-scan-trnas to detect tRNA genes and anvi-run-kegg-kofams to assign KEGG orthologue functions [85].

All individual contigs databases were then combined into a genome storage database with ‘anvi-gen-genomes-storage’, using an external genome file describing the MAGs. The pangenome was generated with ‘anvi-pan-genome’, which uses blastp for amino acid sequence similarity search, and the Markov cluster algorithm to identify gene clusters in the amino acid sequence similarity results, producing a pangenome database that was supplemented with additional metadata layers (anvi-import-misc-data) [86,88]. The inflation parameter was set to 10 to increase the sensitivity of the algorithm, suggested for closely related genomes [86]. ANI was computed between genomes using ‘anvi-compute-genome-similarity’ with pyANI [89], and visualizations were produced with anvi-display-pan.

Within the pangenome, gene clusters were classified into categories based on their distribution across genomes using standard cutoffs: core clusters were defined as present in all 36 genomes; soft-core clusters were present in >95% of genomes [34,36]; shell clusters were present in 2–32 genomes; and singleton clusters were unique to a single genome.

To evaluate the openness of the pangenome and the rate of new gene cluster discovery, we computed rarefaction curves using the programme ‘anvi-compute-rarefaction-curves’ (anvi’o v8-dev build). This tool iteratively samples genomes from the pangenome database and records the cumulative number of gene clusters observed as additional genomes are added. For each sampling scheme, the programme also estimates the size of the conserved core genome. We ran the programme with default parameters, which performs 100 random iterations of genome addition order and reports the resulting average rarefaction curves together with the variance across iterations. The programme reports the estimated Heaps’ law parameters (*κ* and *α*), where the *α* exponent reflects the openness of the pangenome: values of *α*<0.1 indicate a closed pangenome, while *α*>0.1 indicates increasing open pangenomes.

Functional enrichment between the *Mycetohabitans* spp. species was calculated using the anvi’o programme ‘anvi-compute-functional-enrichment-in-pan’ [90] with the functional annotation sources being KOfam and cluster of orthologous groups of proteins (COG20) COG20_FUNCTION and COG20_CATEGORY. This tool tests for functions that occur at significantly different frequencies between predefined genome groups using the Rao score test for equality of proportions. In parallel, KEGG module completeness was estimated for each MAG using ‘anvi-estimate-metabolism’ after KOfam annotation of contig databases. The resulting module-completeness table was used for metabolic enrichment analysis with ‘anvi-compute-metabolic-enrichment’, grouping genomes by species [90]. Modules were considered present at the default anvi’o completeness threshold of 0.75.

### Characterization of the *Apophysomyces*-associated *Mycetohabitans apophysomyceticola* clade

The two *M. apophysomyceticola* MAGs recovered from *Apophysomyces* hosts, provisionally named *M. apophysomyceticola*, were assessed for genome quality using CheckM2 v1.1.0 ‘predict’ against the v1.1.0 reference database [91]. Completeness and contamination estimates were used to ensure that both genomes met accepted high-quality draft MAG standards (≥90% completeness, ≤5% contamination).

Taxonomic assignment was further evaluated with GTDB-Tk ‘classify_wf’ against the current GTDB bacterial reference release 226. This workflow assigns genomes to genus- and species-level clusters based on concatenated single-copy marker phylogenies and ANI to closest reference representatives. As an additional genome-based taxonomic comparison, the two *M. apophysomyceticola* MAGs were uploaded to the Type (Strain) Genome Server (TYGS), a free bioinformatics platform available under https://tygs.dsmz.de, for a whole genome-based taxonomic analysis [92], including introduced methodological updates and features [93]. Nomenclature, synonymy and associated taxonomic annotations were provided by the List of Prokaryotic names with Standing in Nomenclature (LPSN, https://lpsn.dsmz.de, accessed 2025-08-26) [93]. TYGS analysis was subdivided into the following steps. For the phylogenomic inference, all pairwise comparisons among the set of genomes were conducted using GBDP, and accurate intergenomic distances were inferred under the algorithm ‘trimming‘ and distance formula *d_5_* [71]. One hundred distance replicates were calculated each. dDDH values and confidence intervals were calculated using the recommended settings of the GGDC 4.0 [71,93]. The resulting intergenomic distances were used to infer a balanced minimum evolution tree with branch support via FASTME 2.1.6.1 including subtree pruning and regrafting postprocessing [94]. Branch support was inferred from 100 pseudo-bootstrap replicates each. The trees were rooted at the midpoint and visualized with PhyD3 [95]. The type-based species clustering using a 70% dDDH radius around each of the 24 type strains was done as previously described [92]. The resulting groups are shown in Files S4 and S5. Subspecies clustering used a 79% dDDH threshold as previously introduced [96]. Comparisons were carried out between the two *M. apophysomyceticola* MAGs, as well as against the type strains of *M. rhizoxinica* (B1) and *M. endofungorum* (B5) and 20 other automatically selected closely related bacterial strains (File S4). Functional differences relative to other *Mycetohabitans* genomes were assessed using KEGG module content as described above.

## Results

### Public SRA screening identifies *Mycetohabitans*-positive Mucorales datasets

To investigate the prevalence of *Mycetohabitans* sequences in Mucorales genome datasets, 1,696 publicly available raw SRAs with Mucorales metadata, spanning ~50 genera, were screened using Kraken2- and MetaPhlAn4-based taxonomic profiling. This revealed *Mycetohabitans* reads in 46 datasets (File S6), including 13 previously unreported *Mycetohabitans*–Mucorales associations across two genera. Specifically, *Mycetohabitans* was found in the fungal genera *Rhizopus* (*n*=44/541 samples, 8.13% prevalence) and *Apophysomyces* (*n*=2/43 samples, 4.65% prevalence), with varying prevalence at the species level (Table 1, Fig. S2, File S7). Neither MetaPhiAn4 nor Kraken2 identified any sample representing an infection by multiple *Mycetohabitans* species simultaneously. Of the *Mycetohabitans*-positive SRA datasets (File S6), seven samples originated from targeted *Mycetohabitans* sequencing projects (SRR25997369–SRR25997375), which were captured in the initial search based on metadata but lacked corresponding fungal host sequences [49]. These were excluded from additional analysis, beyond geographic plotting. Sequencing datasets originating from *R. microsporus* UCLA 890 (SRR20066946) and *M. rhizoxinica* UCLA 862 (SRR20066947) represent fungal host and bacterial endosymbiont sequencing data recovered from the same clinical case and have been combined in subsequent analyses [37].

**Table 1.** Summary of *Mycetohabitans*-positive fungal sequencing datasets identified in this study, including SRA accession, fungal strain or collection identifier, host taxonomic assignment, isolation source, geographic origin and associated *Mycetohabitans* lineage where resolved. Updated bacterial taxon names marked with an asterisk indicate updated *Mycetohabitans* species assignments based on the analyses performed here. Fungal host names marked with an asterisk indicate records for which the original culture collection or GenBank metadata differed from the taxonomic identification inferred in this study and were therefore updated for downstream analyses. ‘Unknown’ indicates that source or locality information was not available from the associated metadata

SRA accession	Fungal sequencing reference	Previously reported bacterial taxon	Previously reported reference	Bacterial strain ID	Updated bacterial taxon	MAG ID	Fungal host taxon	Fungal strain ID	Isolation source	Geographic location
ERR647685	[55]	*M. rhizoxinica*	[42]	B1 [42]	*M. rhizoxinica*	MAG01	*R. microsporus*	ATCC 62417	Rice seedlings	Japan
SRR11788056	PRJNA628809		This study		Mycetohabitans arrhizicola*	MAG02	*Rhizopus arrhizus*	ZRY 66	Dung	Pakistan
SRR11788061	PRJNA628809		This study		Mycetohabitans arrhizicola*	MAG03	*Rhizopus arrhizus*	XY03822	Soil	South Africa
SRR11788076	PRJNA628809		This study		Mycetohabitans euroasiaticus*	MAG04	*Rhizopus arrhizus*	XY03803	Soil	Philippines
SRR12278013	[47]	*M. endofungorum*	[42]		Mycetohabitans arrhizicola*	MAG05	*Rhizopus arrhizus*	IMI266680	Unknown	Unknown
SRR12354412	[47]	*M. endofungorum*	[42]		Mycetohabitans arrhizicola*	MAG06	*Rhizopus arrhizus*	NRRL A-21579	Rabbit dung	Pakistan
SRR12951099	[44,46]		This study		M. rhizoxinica*	MAG07	R. microsporus*	NRRL 6253	Unknown	Unknown
SRR16016944	[48]	*M. endofungorum*	[48]	EFB03829 [48]	Mycetohabitans arrhizicola*	MAG08	*Rhizopus arrhizus*	XY03829	Soil	Pakistan
SRR16016945	[48]		This study		M. endofungorum*	MAG09	*R. microsporus*	XY03801	Soil	South Africa
SRR20066946/7	[37]	*M. rhizoxinica*	[37]	UCLA_862 [37]	*M. rhizoxinica*	MAG10	*R. microsporus*	UCLA 890	Tracheal aspirate – *Homo sapiens*	USA: Los Angeles
SRR25224693	[54]		This study		Mycetohabitans euroasiaticus*	MAG11	*R. microsporus*	CMC M46	Maxillary sinus – *Homo sapiens*	India: Andhra Pradesh
SRR25224717	[54]		This study		Mycetohabitans euroasiaticus*	MAG12	*R. microsporus*	CMC M24	Maxillary sinus – *Homo sapiens*	India: Tamil Nadu
SRR25224725	[54]		This study		Mycetohabitans homothallicola*	MAG13	*Rhizopus homothallicus*	CMC M17	Middle turbinate – *Homo sapiens*	India: Tamil Nadu
SRR7686240	[44,46]	*Mycetohabitans* sp.	[42]		Mycetohabitans apophysomyceticola*	MAG14	*Apophysomyces* sp.	BC1015	Unknown	USA: Oklahoma
SRR7686242	[44,46]	*Mycetohabitans* sp.	[42]		Mycetohabitans apophysomyceticola*	MAG15	*Apophysomyces* sp.	BC1034	Unknown	USA: Oklahoma
SRR8485126	[44,46]	*M. endofungorum*	[42]	B75 [50]	Mycetohabitans arrhizicola*	MAG16	Rhizopus arrhizus*	NRRL 66675	Rabbit dung	Pakistan
SRR8845249	[56]		This study		Mycetohabitans australianus*	MAG17	*R. microsporus*	B07585	*Homo sapiens*	USA
SRR8845250	[56]		This study		M. rhizoxinica*	MAG18	*R. microsporus*	B07386	*Homo sapiens*	USA
SRR8845252	[56]		This study		M. rhizoxinica*	MAG19	*R. microsporus*	B08956	*Homo sapiens*	USA: Georgia
SRR9029062	[44,46]	*M. rhizoxinica*	[42]	B82 [50]	*M. rhizoxinica*	MAG20	*Rhizopus arrhizus*	NRRL 2582	Hospital mattress	USA: Ohio
SRR9029124	[44,46]	*M. rhizoxinica*	[42]	B50 [50]	*M. rhizoxinica*	MAG21	*R. microsporus*	NRRL 5550	Unknown	USA: New York
SRR9029145	[44,46]	*M. rhizoxinica*	[42]	B48 [50]	*M. rhizoxinica*	MAG22	*R. microsporus*	NRRL 5548	Hospital	USA: Illinois
SRR9029155	[44,46]	*Mycetohabitans* sp.	[42]	B46 [50]	Mycetohabitans cryptica*	MAG23	*R. microsporus*	NRRL 5546	Soil	Brazil
SRR9029156	[44,46]	*M. rhizoxinica*	[42]		*M. rhizoxinica*	MAG24	*R. microsporus*	NRRL 5547	Soil	Philippines
SRR9029401	[44,46]	*M. endofungorum*	[42]	B53 [50]	Mycetohabitans arrhizicola*	MAG25	*R. microsporus*	NRRL 5553	Soil	South Africa
SRR9029403	[44,46]	*M. endofungorum*	[42]		Mycetohabitans arrhizicola*	MAG26	Rhizopus arrhizus*	NRRL 5554	Soil	South Africa
SRR9650540	[44,46]	*M. rhizoxinica*	[42]	B514 [50]	*M. rhizoxinica*	MAG27	R. microsporus*	NRRL 1514	Decaying plant matter	Unknown
SRR9650546	[44,46]	*M. rhizoxinica*	[42]		*M. rhizoxinica*	MAG28	R. microsporus*	NRRL 2934	Pack rat dung	USA: California
SRR9650549	[44,46]	*M. rhizoxinica*	[42]		*M. rhizoxinica*	MAG29	Rhizopus arrhizus*	NRRL 3373	Unknown	Unknown
SRR9650585	[44,46]		This study		M. endofungorum*	MAG30	R. microsporus*	NRRL 66564	Soil	South Africa
SRR9712532	[44,46]	*M. rhizoxinica*	[42]	B52 [50]	*M. rhizoxinica*	MAG31	*R. microsporus*	NRRL 5552	Creek sediment	USA: Ohio
SRR9712541	[44,46]	*M. endofungorum*	[42]	B51 [50]	Mycetohabitans euroasiaticus*	MAG32	*R. microsporus*	NRRL 5551	Unknown	Philippines
SRR9720186	[44,46]	*M. rhizoxinica*	[42]	B58 [50]	*M. rhizoxinica*	MAG33	*R. microsporus*	NRRL 5558	Corn	USA
SRR9720220	[44,46]	*M. rhizoxinica*	[42]		*M. rhizoxinica*	MAG34	R. microsporus*	NRRL A-11791	Unknown	Unknown
SRR9720297	[44,46]	*M. rhizoxinica*	[42]		*M. rhizoxinica*	MAG35	*R. microsporus*	NRRL A-26124	Unknown	Unknown
SRR9720300	[44,46]	*M. rhizoxinica*	[42]	B23 [50]	Mycetohabitans discreta*	MAG36	*Rhizopus arrhizus*	NRRL 62023	Corn	USA: Nebraska
SRR9720307	[44,46]	*M. endofungorum*	[42]	B29 [50]	*M. endofungorum*	MAG37	*R. microsporus*	NRRL 13129	Unknown	Unknown
SRR9720377	[44,46]		This study		Mycetohabitans arrhizicola*	MAG38	*Rhizopus arrhizus*	NRRL A-11376	Unknown	Unknown

### *Mycetohabitans* endosymbiont genome reconstruction and quality assessment

To better understand whether *Mycetohabitans* sequences represented true endosymbionts or simply contaminating reads, we attempted to assemble the bacterial genomes. Metagenomic binning and refinement yielded 38 *Mycetohabitans* metagenomic-assembled genomes (MAGs), spanning a range of completeness (Table 2). Of these, 34 MAGs were classified as higher quality, characterized by recovery of all 124 bacterial BUSCO single-copy orthologues, and with genome lengths between 3.3 and 3.7 Mbp and GC contents of ~60–61%. Two MAGs (MAG05 and MAG06) were incomplete, with between 79 and 103 complete BUSCO genes and reduced genome sizes (2.6–3.2 Mbp). A further two MAGs (MAG29 and MAG35) were of poor quality, exhibiting <10% complete BUSCOs with severe fragmentation (<2 Mbp total length and high proportions of missing/fragmented orthologues), and were therefore excluded from downstream analyses.

**Table 2.** Summary of MAGs generated from endosymbiont contigs, including sample accession, taxonomic assignment, assembly size, contiguity and completeness metrics

MAG ID	Bacterial MAG SRA origin	Bacterial species	# contig	Largest contig (bp)	Total length (bp)	GC (%)	Gene count	N50	N90	auN	L50	L90	# N's per 100 kbp	BUSCO complete count	BUSCO single count	BUSCO duplicated count	BUSCO fragmented count	BUSCO missing count	BUSCO total count
MAG01	ERR647685	*M. rhizoxinica*	206	186,637	3,617,497	60.74	3,344	77,714	13,843	77,862.5	17	53	0	124	124	0	0	0	124
MAG02	SRR11788056	*Mycetohabitans arrhizicola*	153	200,722	3,429,698	60.85	3,155	81,563	15,215	85,768.4	14	55	0	124	124	0	0	0	124
MAG03	SRR11788061	*Mycetohabitans arrhizicola*	148	239,460	3,484,487	60.81	3,200	77,446	15,224	91,534.6	14	51	0	124	123	1	0	0	124
MAG04	SRR11788076	*Mycetohabitans euroasiaticus*	68	459,748	3,521,807	60.58	3,185	171,308	44,618	206,806.3	6	25	0	124	124	0	0	0	124
MAG05	SRR12278013	*Mycetohabitans arrhizicola*	1,711	9,526	2,598,975	61.37	3,604	1,850	772	2,263.5	443	1,302	0	79	78	1	32	13	124
MAG06	SRR12354412	*Mycetohabitans arrhizicola*	1,045	24,043	3,157,256	60.99	3,596	4,477	1,440	5,606	209	704	0	103	103	0	18	3	124
MAG07	SRR12951099	*M. rhizoxinica*	56	276,031	3,506,927	60.99	3,114	150,076	56,467	151,517.1	9	23	0	124	124	0	0	0	124
MAG08	SRR16016944	*Mycetohabitans arrhizicola*	151	200,722	3,430,428	60.85	3,153	81,563	15,215	85,784.8	14	55	0	124	124	0	0	0	124
MAG09	SRR16016945	*M. endofungorum*	48	994,910	3,362,766	61.12	3,061	321,097	52,237	442,429.6	3	13	0	124	124	0	0	0	124
MAG10	SRR20066946/7	*M. rhizoxinica*	74	469,633	3,573,995	60.9	3,173	183,135	45,224	204,245.3	7	23	0	124	124	0	0	0	124
MAG11	SRR25224693	*Mycetohabitans euroasiaticus*	135	262,772	3,669,156	60.63	3,283	126,942	22,147	118,568.1	11	39	0	124	124	0	0	0	124
MAG12	SRR25224717	*Mycetohabitans euroasiaticus*	109	345,505	3,516,646	60.64	3,115	94,668	21,656	127,836.7	11	37	0	124	124	0	0	0	124
MAG13	SRR25224725	*Mycetohabitans homothallicola*	91	320,811	3,355,502	62.93	3,059	115,676	26,362	133,487.8	10	32	0	122	122	0	0	2	124
MAG14	SRR7686240	*M. apophysomyceticola*	538	23,618	1,683,483	63.21	1,962	4,561	1,504	5,310.1	120	368	0	99	98	1	19	6	124
MAG15	SRR7686242	*M. apophysomyceticola*	47	241,984	2,045,747	62.7	1,894	120,886	37,612	128,205.5	6	19	0	121	121	0	1	2	124
MAG16	SRR8485126	*Mycetohabitans arrhizicola*	876	28,111	3,288,452	60.93	3,567	6,159	1,755	7,420.7	165	558	0	103	103	0	16	5	124
MAG17	SRR8845249	*Mycetohabitans australianus*	31	843,296	3,328,361	61.12	2,963	462,047	111,337	425,821.4	3	11	0	124	124	0	0	0	124
MAG18	SRR8845250	*M. rhizoxinica*	96	538,081	3,647,527	60.7	3,289	138,836	31,878	236,313.5	6	26	0	124	124	0	0	0	124
MAG19	SRR8845252	*M. rhizoxinica*	97	444,434	3,662,714	60.73	3,370	160,500	24,128	168,802.1	9	28	0	124	124	0	0	0	124
MAG20	SRR9029062	*M. rhizoxinica*	89	281,496	3,342,728	61.04	3,120	85,870	27,545	116,283.8	11	37	0	124	124	0	0	0	124
MAG21	SRR9029124	*M. rhizoxinica*	45	610,619	3,490,640	60.96	3,109	169,617	51,889	248,570.3	6	20	0	124	124	0	0	0	124
MAG22	SRR9029145	*M. rhizoxinica*	83	284,578	3,77,2308	60.81	3,405	143,212	44,624	146,387.6	10	28	0	124	113	11	0	0	124
MAG23	SRR9029155	*Mycetohabitans cryptica*	75	319,995	3,317,950	61.06	3,078	86,439	26,655	105,579.7	13	39	0	124	124	0	0	0	124
MAG24	SRR9029156	*M. rhizoxinica*	71	542,141	3,426,381	61.1	3,102	143,374	33,206	229,071.5	6	24	0	124	124	0	0	0	124
MAG25	SRR9029401	*Mycetohabitans arrhizicola*	98	305,230	3,641,185	60.84	3,265	86,147	30,255	112,944	13	40	0	124	124	0	0	0	124
MAG26	SRR9029403	*Mycetohabitans arrhizicola*	164	167,722	3,454,114	60.81	3,182	59,085	13,492	63,277.2	20	68	0	124	124	0	0	0	124
MAG27	SRR9650540	*M. rhizoxinica*	900	42,384	3,572,073	60.75	3,841	6,715	1,711	8,780.8	161	563	0	110	110	0	12	2	124
MAG28	SRR9650546	*M. rhizoxinica*	106	375,762	3,507,941	61.09	3,156	104,598	34,047	136,241.7	10	31	0	123	123	0	0	1	124
MAG29	SRR9650549	*M. rhizoxinica*	462	2,765	330,964	61.22	–	697	523	798.5	177	398	0	5	5	0	12	107	124
MAG30	SRR9650585	*M. endofungorum*	109	317,877	3,351,310	61.13	3,108	119,908	22,180	126,697.5	10	36	0	124	124	0	0	0	124
MAG31	SRR9712532	*M. rhizoxinica*	92	530,011	3,508,128	60.95	3,217	118,546	30,997	192,270.9	8	32	0	124	124	0	0	0	124
MAG32	SRR9712541	*Mycetohabitans euroasiaticus*	108	811,375	3,618,100	60.53	3,293	154,040	18,293	278,804.7	6	30	0	124	124	0	0	0	124
MAG33	SRR9720186	*M. rhizoxinica*	54	445,358	3,602,372	60.85	3,255	154,841	54,894	201,389.9	7	23	0	124	124	0	0	0	124
MAG34	SRR9720220	*M. rhizoxinica*	269	96,979	3,305,558	61.27	3,158	24,150	5,362	30,038.3	40	143	0	123	123	0	1	0	124
MAG35	SRR9720297	*M. rhizoxinica*	1,641	3,940	1,556,019	61.15	–	989	592	1,157.4	538	1,354	0	41	41	0	41	42	124
MAG36	SRR9720300	*Mycetohabitans discreta*	187	218,427	3,525,908	60.78	3,259	37,773	10,662	53,130.5	28	91	0	123	123	0	1	0	124
MAG37	SRR9720307	*M. endofungorum*	55	747,654	3,305,516	61.25	3,013	206,315	55,453	297,321.6	5	15	0	124	124	0	0	0	124
MAG38	SRR9720377	*Mycetohabitans arrhizicola*	318	91,266	3,260,833	61.02	3,127	19,588	4,986	28,392.3	45	165	0	122	122	0	2	0	124

On average, the MAGs contained ~3.55 Mbp across ~150 contigs, with *N*_50_ values ranging from 60 to 170 kb. BUSCO analysis confirmed the presence of all 124 complete orthologues in nearly all cases, consistent with the expected gene content of *Mycetohabitans* reference strains (119–124/124 BUSCO genes identified). The assembled MAG sizes were also broadly consistent with the published *Mycetohabitans* complete genomes (3.35–3.80 Mb, File S2).

Read recruitment provided further support that the recovered bacterial contig sets represented coherent genome assemblies. Across all 38 bacterial bins, mapped reads covered almost the entirety of the recovered bacterial contig sets, with genome-wide breadth of coverage ranging from ~99.97–100% and a median of ~99.999% (File S8). This indicates that bacterial reads were distributed across the reconstructed *Mycetohabitans* assemblies rather than being restricted to a small number of highly abundant contigs. Mean sequencing depth varied substantially among datasets, consistent with variable bacterial abundance and sequencing effort among public fungal libraries. Together, the recovery of *Mycetohabitans*-sized genome assemblies, bacterial BUSCO profiles, expected GC content, broad read-recruitment coverage and contig-level taxonomic support indicate that these datasets contain coherent *Mycetohabitans* genomes rather than sporadic bacterial contaminants. However, because intracellular localization cannot be confirmed from sequencing data alone, these records are conservatively interpreted as genome-supported putative *Mycetohabitans* endosymbiont associations.

### Genome-based species delimitation resolves nine *Mycetohabitans* species-level clusters

**Fig. 1.**
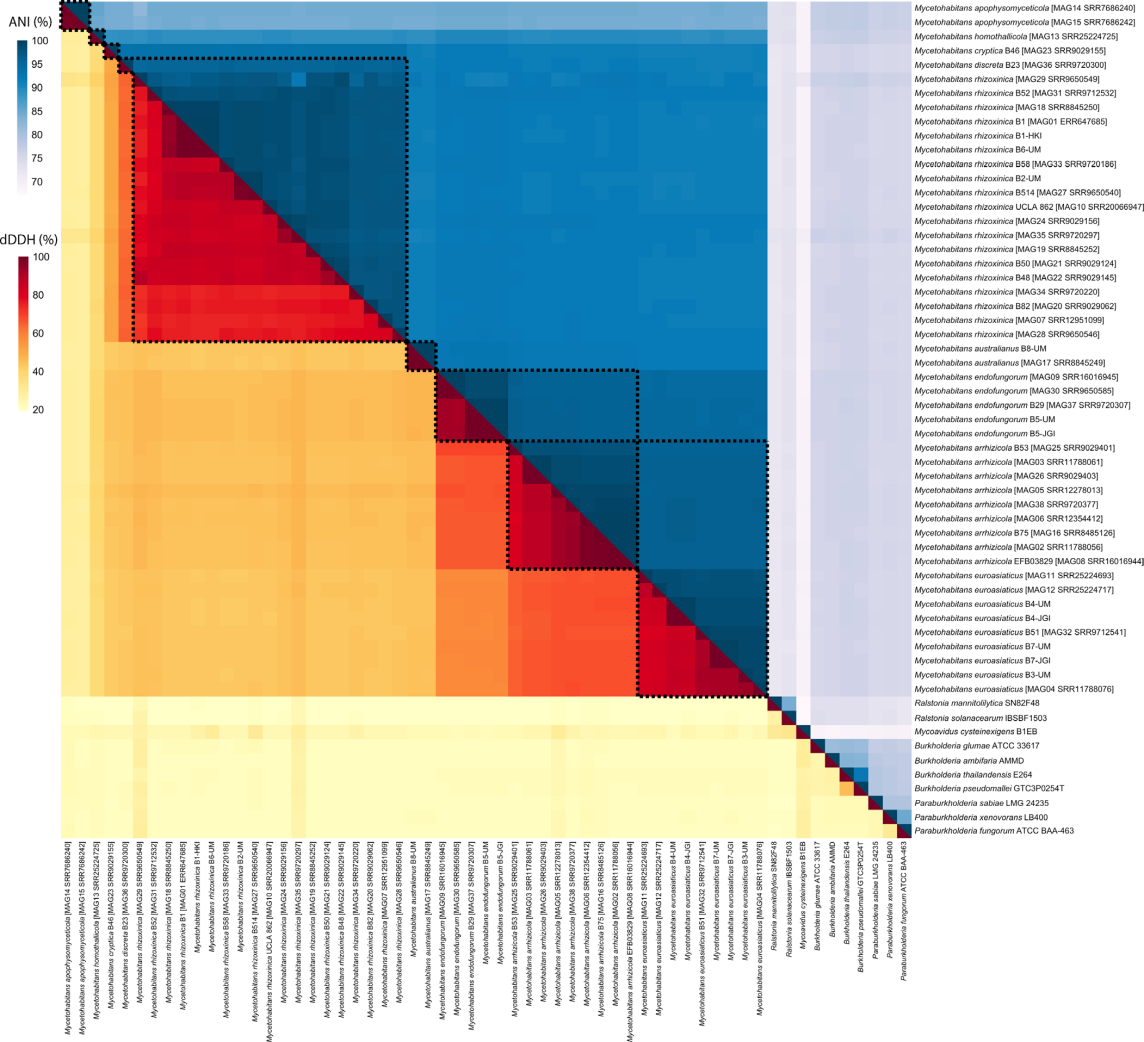
Genome-based species delimitation of *Mycetohabitans* and *Burkholderia sensu lato* genomes and MAGs using ANI and dDDH. Pairwise genomic similarity among all analysed *Mycetohabitans* and *Burkholderia* sensu lato genomes and metagenome-assembled genomes (MAGs), shown as a combined triangular heatmap. The upper-right matrix displays average nucleotide identity values (ANI), whereas the lower-left matrix displays digital DNA–DNA hybridisation values (dDDH). Genomes are arranged by genomic relatedness, revealing nine species-level clusters based on accepted cut-offs of >95% ANI and >70% dDDH; species-level clusters are indicated with dotted lines. Proposed species-level lineages are labelled as *M. apophysomyceticola*, *M. arrhizicola*, *M. homothallicola*, *M. cryptica*, and *M. discreta*. Labels include strain names or MAG identifiers, with source SRA accessions provided for MAG-derived assemblies.

Genome-based species delimitation using ANI and dDDH resolved the *Mycetohabitans* dataset into nine species-level clusters (ANI >95%; dDDH >70%; Fig. 1). Of these, four correspond to previously described species-level groups, two represent additional novel multi-isolate clusters and three are novel singletons represented by individual genomes [23]. In order of occupancy, Cluster 1 comprised *M. rhizoxinica* strains B1, B6 and B2 together with 16 MAGs recovered from fungal sequencing datasets. Cluster 2 comprised *Mycetohabitans euroasiaticus* strains B3, B4 and B7 together with four recovered MAGs. Cluster 3 is a novel multi-isolate cluster compromising nine MAGs, provisionally designated *Mycetohabitans arrhizicola* based on its fungal host association. Cluster 4 comprised *M. endofungorum* strain B5 together with three recovered MAGs. Cluster 5 comprised *Mycetohabitans australianus* strain B8 and one MAG from a Brazilian fungal isolate. Cluster 6 was a novel multi-isolate cluster compromising two MAGs from *Apophysomyces* hosts, provisionally designated *M. apophysomyceticola*. Clusters 7, 8 and 9 are each represented by a single MAG and are here treated as distinct singleton lineages (Fig. 1, Table 1). Together, these results indicate substantially greater species-level diversity within the sampled *Mycetohabitans* genomes than previously recognized.

### Geographic distribution and host identity of *Mycetohabitans*-positive datasets

**Fig. 2.**
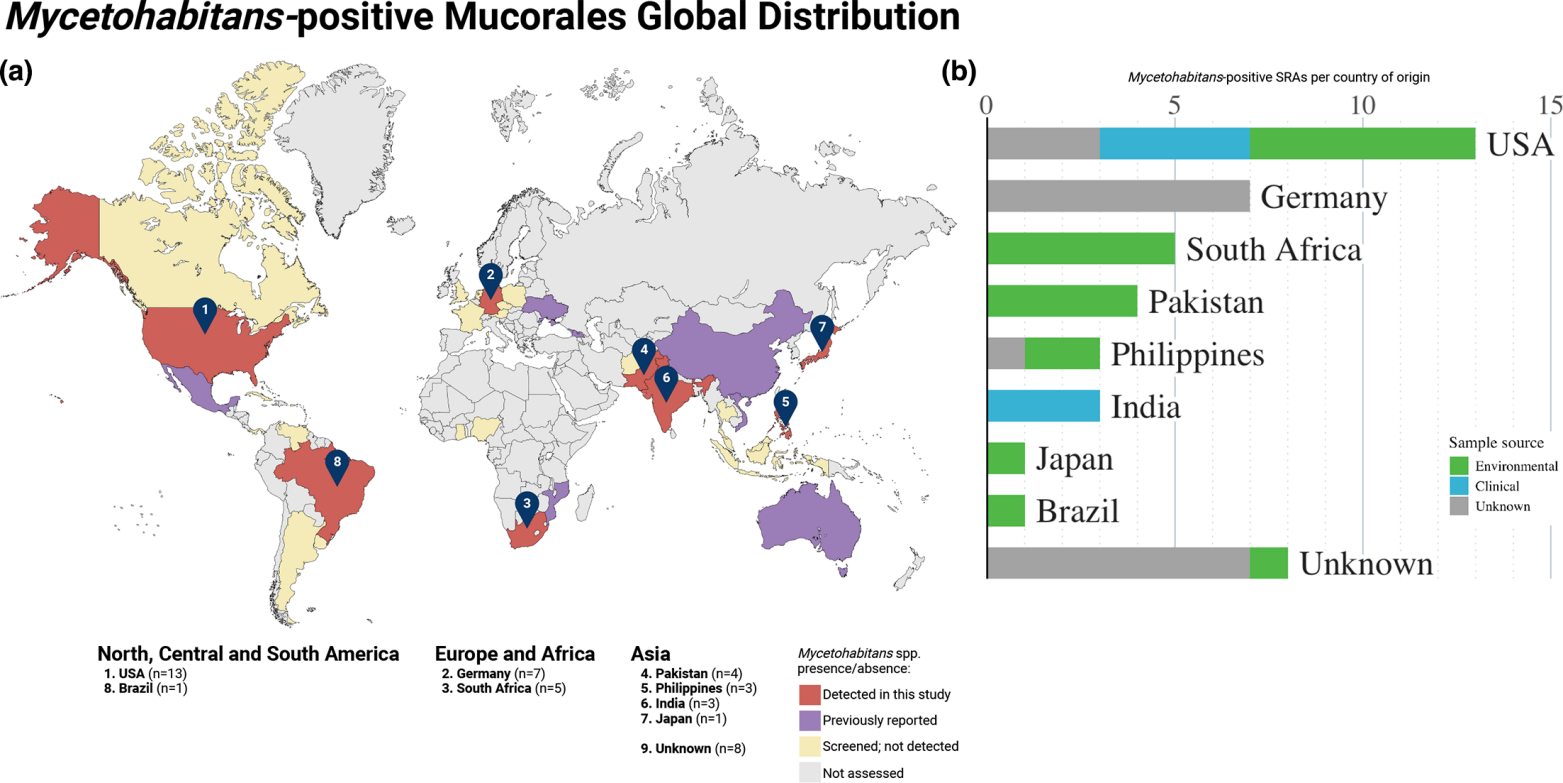
Geographic distribution of *Mycetohabitans*-positive SRA datasets. (**a**) World map showing the countries of origin for Mucorales fungal isolates identified with *Mycetohabitans* signals in their WGS. Countries are coloured according to evidence for *Mycetohabitans*-associated fungal samples. ‘Detected in this study’ indicates countries with *Mycetohabitans*-positive datasets identified through our screening of public sequencing data. ‘Previously reported’ indicates countries with *Mycetohabitans* associations reported in the literature. ‘Screened; not detected’ indicates countries represented in the datasets analysed here for which no *Mycetohabitans*-positive dataset was identified; this should not be interpreted as evidence of true absence. Countries with no suitable public data available for screening are shown separately as ‘not assessed’. (**b**) Bar plot summarizing the number of *Mycetohabitans*-positive accessions per country and their collection source either ‘Environmental’, ‘Clinical’ or ‘Unknown’. Positive datasets lacking associated geographic metadata are labelled ‘Unknown.’ Country-level totals of datasets analysed, positive hits and calculated prevalence are provided in File S4.

The distribution of *Mycetohabitans*-positive SRAs varied widely across geographic origins (Fig. 2a, File S9), with *Mycetohabitans*-positive SRAs identified in isolates from North and South America (USA, *n*=15/343; 4.4%; Brazil, *n*=1/1; 100%), Europe (Germany, *n*=7/12; 58%), Africa (South Africa, *n*=5/7; 71%) and Asia (Pakistan, *n*=4/11; 36%; India, *n*=3/106; 2.8%; the Philippines, *n*=3/4; 75%; and Japan, *n*=1/2; 50%). Isolates originating from unknown locations were rarely positive (*n*=8/1074; 0.7%). No positives were detected from 20 additional countries, including Australia (*n*=34), Argentina (*n*=24), China (*n*=11), Poland (*n*=11) and several others with smaller sample numbers (Afghanistan, Antarctica, Austria, England, Cuba, Canada, Bahamas, Indonesia, Ghana, Malaysia, Mexico, Netherlands, Puerto Rico, Thailand, Uruguay and Venezuela; *n*=72). These findings indicate that while *Mycetohabitans* occur globally, prevalence is highly heterogeneous, and detection is likely constrained by limited sampling and underrepresentation of infected isolates in public datasets.

**Fig. 3.**
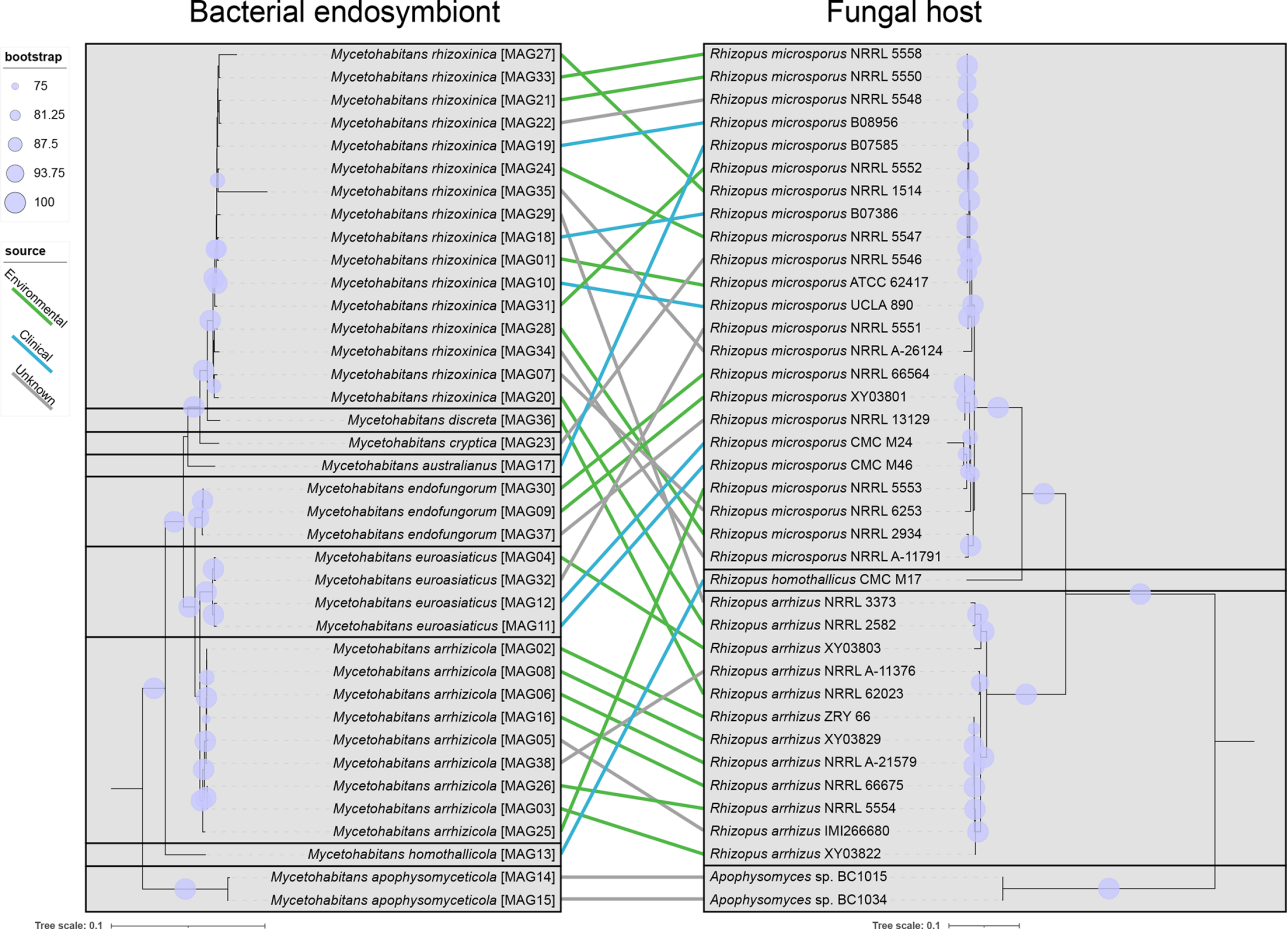
Tanglegram of identified bacterial endosymbionts and their fungal hosts. Maximum-likelihood phylogenies were reconstructed for (left) *Mycetohabitans* bacterial endosymbiont MAGs and (right) their associated mucoralean fungal hosts. Both trees show bootstrap support values from 1,000 replicates. Connecting lines linking each fungal host to its identified bacterial symbiont are colour coded based on isolation source: Green, environmental; blue, clinical human infection; grey, unknown.

Fungal datasets were globally distributed and included both environmental and clinical samples (Fig. 2b, Table 1). Fungal host identification was verified only for SRA datasets that exhibited a bacterial sequence signature, by comparing metadata with ITS sequence, host genome assembly reconstruction and whole-genome phylogenetics (Fig. 3, Table S10). The re-identification revealed eight samples that were either mislabelled in fungal collection catalogues or misannotated in GenBank, representing 20.5% of the analysed bacterial-positive fungal sequencing datasets (*n*=8/39; indicated with an asterisk in Table 1; File S10).

At the fungal host level, reads consistent with *Mycetohabitans* species were identified in three fungal species (*R. microsporus*, *Rhizopus arrhizus* and *Rhizopus homothallicus*) and one additional host taxon identified only to genus level (*Apophysomyces* sp.). Fig. 3 presents a tanglegram linking bacterial and fungal species phylogenies. Of the Mucorales species identified as positive for *Mycetohabitans* infection, three have previously been reported to harbour endosymbionts: *R. microsporus* hosting *M. endofungorum* and *M. rhizoxinica*, *R. arrhizus* hosting *M. endofungorum* and *Apophysomyces* sp. hosting an unidentified *Mycetohabitans* sp. [21,48]. Our updated species-level delimitation resolves this previously broad *M. endofungorum–R. arrhizus* association into a distinct *R. arrhizus*-associated lineage. Specifically, MAGs from *R. arrhizus* datasets that were previously reported as *M. endofungorum* clustered instead within a novel species-level group, here provisionally designated *M. arrhizicola*. Under this revised framework, *M. endofungorum* was recovered only from *R. microsporus* datasets, suggesting that earlier reports of *M. endofungorum* in *R. arrhizus* reflected the limited taxonomic resolution available before recognition of this additional *Mycetohabitans* species-level cluster. The endosymbiont of *R. homothallicus* was resolved as a novel singleton species-level lineage, here provisionally designated *Mycetohabitans homothallicola*.

### Updated species delimitation reveals non-random *Mycetohabitans* host associations

Prevalence of *Mycetohabitans* infection was broadly similar between the two dominant hosts, with positives detected in 12/152 *R*. *arrhizus* samples (7.9%) and 23/248 *R*. *microsporus* samples (9.3%) across both environmental and clinical isolates. However, updated species-level delimitation revealed non-random host–symbiont pairings across the full set of recovered associations. A fungal species-bacterial species contingency table showed significant departure from independence (Pearson’s chi-squared test with simulated *P*-value, *X*²=97.669, *P*=9.999×10⁻⁵), indicating that *Mycetohabitans* species are not randomly distributed across fungal hosts (Figs 3 and S3). Consistent with this, ParaFit analysis detected significant global congruence between fungal host and bacterial symbiont phylogenies (ParaFitGlobal=0.0234, *P*=0.0003; 9,999 permutations). After FDR correction, the two *Apophysomyces–M. apophysomyceticola* links remained individually significant, whereas most other individual links were not significant or were discordant, suggesting that host–symbiont structure is strongest for the *Apophysomyces*-associated lineage but that broader host associations are also non-random (Fig. 3, File S11).

Focusing on known clinical isolates, *Mycetohabitans*-positive samples derived from human infections were almost exclusively associated with *R. microsporus* (*n*=6/7), including sinus, tracheal aspirate and other clinical specimens from the USA and India (Fig. 2b, Table 1). Of these, three were identified as *M. rhizoxinica*, two as *M. euroasiaticus* and one as *M. australianus*. Additionally, a single *M. homothallicola* endosymbiont was detected in *R. homothallicus*. No *Mycetohabitans* sequences were detected among clinical *R. arrhizus* isolates, despite the frequent identification of *M. arrhizicola* in *R. arrhizus* from environmental samples and occasional *M. euroasiaticus* and *M. rhizoxinica* associations also identified with *R. arrhizus* (Fig. 3). As noted above, a substantial fraction of public Mucorales datasets are misidentified at both the species and genus levels. Therefore, it is possible that some of the endosymbiont-negative samples are assigned to the wrong host species.

### Pangenome analysis of *Mycetohabitans* endosymbiont genomes reveals an open accessory genome and lineage-associated functional variation

Models of endosymbiont evolution suggest that endosymbiont genomes progressively lose dispensable genes as they become dependent on their hosts for basic biosynthetic pathways [97]. At the same time, endosymbionts can selectively retain or become enriched for factors that advantage the holobiont, such as biosynthetic gene clusters which produce toxins that enable holobiont evasion of microbial predators [8,25]. As these processes generate pronounced variation in gene content, a pangenome framework is essential for resolving which functions are universally conserved versus those that reflect lineage-specific adaptations. We therefore constructed an anvi’o pangenome from 36 metagenome assembled genome assemblies (MAGs) of *Mycetohabitans* sp.

**Fig. 4.**
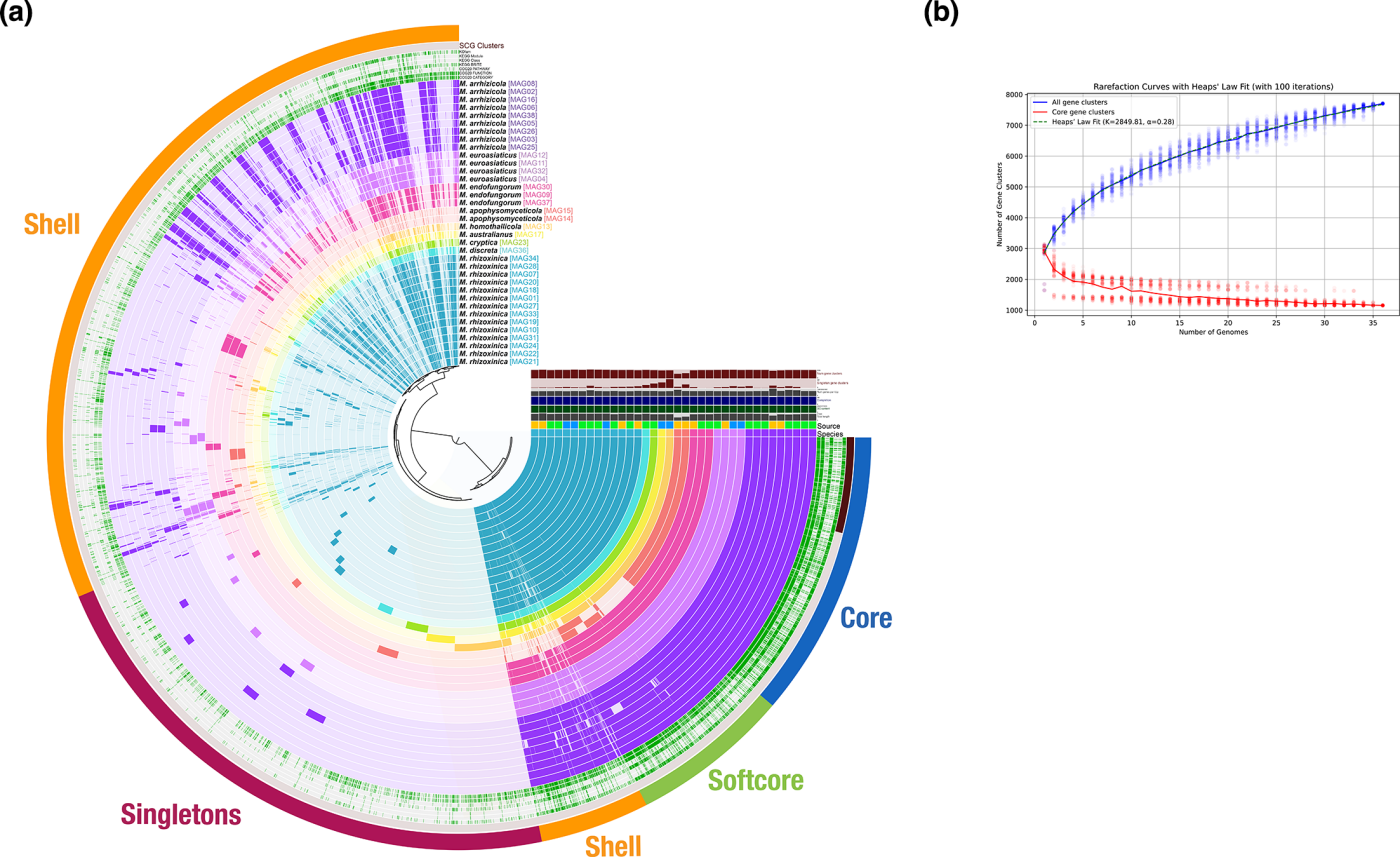
Pangenome structure and diversity of the 36 high-quality *Mycetohabitans* MAGs. (**a**) Pangenome of 36 high-quality MAGs. The genomes are coloured to represent their *Mycetohabitans* species, respectively, where gene clusters are arranged based on gene cluster presence/absence. Radiating out from these layers are the annotations from cluster of orthologous groups of proteins (COG20) Category, COG20 Function, COG20 Pathway, KEGG Brite, KEGG Class, KEGG Module and KOfam. The penultimate outermost dark red layer highlights single-copy core gene clusters found in all 36 genomes. The outermost layer indicated pangenome occupancy categories: core (present in all genomes, blue), softcore (present in >95%, green), shell (intermediate frequency, orange) and singleton (present in only one genome, dark red). MAG statistics are represented as bar charts in descending order: number of gene clusters present, number of singleton gene clusters present, number of genes per kbp, completion, GC content, total length and isolation source (environmental, green; clinical human infection, blue; unknown, orange). (**b**) Rarefaction curves with Heaps’ law fit for the *Mycetohabitans* pangenome. Rarefaction analysis was performed with 100 random genome addition iterations across 36 genomes. Curves show the accumulation of gene clusters for the pangenome (blue) and the core genome (red), with shaded areas indicating variation across iterations. The estimated Heaps’ law exponent (*α*=0.2765) indicates an open pan-genome.

Across all isolates, we identified 7,706 gene clusters comprising 113,762 gene calls. Based on occupancy across the 36 genomes, these gene clusters were further categorized as core (shared by all 36 genomes), softcore (present in 34–35 genomes), shell (present in 2–33 genomes) and singleton (present in 1 genome only). By number of clusters, the pangenome comprised 1,156 core gene clusters (15.0%), 295 softcore gene clusters (3.8%), 4,002 shell gene clusters (51.9%) and 2,253 singleton gene clusters (29.2%) (Fig. 4a). Thus, most distinct gene clusters belonged to the accessory genome, especially the shell and singleton fractions

When considering the total number of genes mapped to these clusters (111,851 genes), the distribution differed: 39.2% mapped to core gene clusters, 9.6% to softcore gene clusters, 49.1% to shell gene clusters and 2.0% to singleton gene clusters. This indicates that although core gene clusters make up a relatively small fraction of all distinct clusters, they account for a substantial proportion of total gene content because they are present in nearly all genomes. In contrast, singleton gene clusters represent many unique clusters but only a small fraction of total mapped genes because each occurs only once.

Each genome assembly had on average 2,887 annotated gene clusters (range 1,645–3136), of which 1,731 on average were accessory (range 489–1,980; present in 2–35 genomes), representing a mean 59.3% of the gene content. Singleton burden averaged 63 gene clusters per genome (range 0–367). The occupancy distribution was right-skewed, with 2,253 singleton gene clusters and 1,156 core gene clusters conserved across all 36 genomes, showing many rare clusters and a comparatively small core genome. Rarefaction analyses indicated an open pangenome [Heaps’ law exponent (*α*)=0.2765], indicating more gene clusters are still discovered upon additional genomes being added (Fig. 4b).

Together, these results show that *Mycetohabitans* genomes retain a stable conserved core while also harbouring a large and diverse accessory gene pool. Biologically, this pattern is consistent with lineage-specific gene gain, loss and turnover, likely reflecting adaptation to different host associations and evolutionary trajectories. This pangenome structure provides the framework for identifying which biological processes have been consistently retained, repeatedly lost or uniquely expanded across endosymbiont lineages.

Across the 36 MAGs with revised species-level assignments, anvi’o functional enrichment analysis was carried out using species as the categorical variable. The dataset comprised nine species-level lineages: *M. rhizoxinica* (*n*=14), *M. arrhizicola* (*n*=9), *M. euroasiaticus* (*n*=4), *M. endofungorum* (*n*=3), *M. apophysomyceticola* (*n*=2) and four singleton lineages, *M. australianus*, *Mycetohabitans cryptica*, *Mycetohabitans discreta* and *M. homothallicola* (*n*=1 each). Due to several lineages being represented by few genomes, enrichments in low-sample lineages were interpreted as lineage-associated patterns rather than definitive species-level signatures. The *M. apophysomyceticola* MAGs were notably reduced relative to other *Mycetohabitans* genomes and are therefore considered separately below as a distinct case of genome reduction rather than interpreted solely through the broader functional enrichment framework.

KEGG module enrichment revealed functional differentiation across *Mycetohabitans* species-level lineages (File S12). Modules with variable presence/absence across the *Mycetohabitans* MAGs included functions associated with central carbon metabolism, cell-envelope precursor biosynthesis, amino acid and nucleotide degradation, *β*-oxidation, ketone-body metabolism, nitrate metabolism, aromatic compound degradation, cofactor biosynthesis, secretion systems and antimicrobial resistance-associated functions. Modules enriched in *M. rhizoxinica* included *β*-oxidation/leucine degradation, pyrimidine and guanine degradation, tyrosine degradation, dissimilatory nitrate reduction/denitrification, semi-phosphorylative Entner–Doudoroff metabolism, d-galacturonate/d-glucuronate degradation, biotin biosynthesis and several aromatic-compound degradation pathways. However, most of these enrichments were also shared with closely related lineages, particularly the singleton species *M. cryptica*, *M. discreta*, *M. australianus* and/or *M. apophysomyceticola*. This is consistent with a model in which these genes have been lost in the *M. endofungorum-*related lineages. Although there is not statistical support for association with individual species within *M*. endofungorum-lineages (*M. endofungorum*, *M. arrhizicola* and *M. euroasiaticus*) due to a lack of power, when considered as a group, there appears to be enrichment of virulence and multi-drug resistance-related genes (T1SS/efflux pumps) by KEGG functional analysis.

COG20 functional enrichment recovered complementary gene cluster-level variation (File S13). Variation included transporters, secretion- and cell-envelope-associated proteins, oxidative stress and metal resistance functions, plasmid/mobile element-associated proteins, amino acid and lipid metabolism enzymes and secondary metabolism-associated domains. Several strong COG20 enrichments were associated with singleton or low-sample lineages, including arsenite efflux and tryptophan metabolism in *M. cryptica*, aminopeptidase and non-ribosomal peptide synthetase-associated functions in *M. discreta* and plasmid replication, iron transport, copper binding, lipid/polyketide synthase-associated domains and acyl-CoA metabolism in *M. australianus*.

KOfam enrichment provided complementary gene-level resolution of these functional patterns (File S14). Varied KOfam annotations included genes involved in central metabolism, sulphate/thiosulphate transport, nitrogen regulation, type III secretion, type IV pilus assembly, cell-envelope modification, metal transport, DNA repair and stress-associated functions. More restricted enrichments included tryptophan 2,3-dioxygenase, kynureninase, arsenite transport and ArsR-family arsenite-responsive regulation in *M. cryptica* and pyrroloquinoline quinone biosynthesis, type IV secretion, phospholipase C, nickel/cobalt transport and Cu(I)/Ag(I) efflux functions in *M. homothallicola*. Because these two lineages were each represented by a single genome, these patterns should be interpreted as lineage-associated signals requiring confirmation with additional genomes.

Metabolic module enrichment based on reconstructed module presence further supported a mosaic distribution of functional capacity across the revised species assignments. Broadly, varied modules included leucine biosynthesis, the ornithine-ammonia cycle, the oxidative pentose phosphate pathway and dTDP-l-rhamnose biosynthesis. More restricted module gains included tryptophan metabolism and NAD biosynthesis in *M. cryptica*, MexJK-OprM multidrug efflux in *M. endofungorum*, pyrimidine degradation and the semi-phosphorylative Entner–Doudoroff pathway in *M. rhizoxinica*, *M. cryptica* and *M. discreta* and homoprotocatechuate degradation across *M. rhizoxinica*, *M. homothallicola*, *M. australianus*, *M. cryptica* and *M. discreta* (File S15).

Together, the KEGG module, metabolic module, COG20 and KOfam analyses indicate that functional diversity within *Mycetohabitans* is distributed across multiple species-level lineages. Rather than resolving into a single lineage-specific functional profile, the enrichment patterns reveal a mosaic of broadly shared metabolic capacities, variably retained catabolic pathways, secretion and cell-envelope-associated systems, metal and antimicrobial resistance functions and more restricted lineage-associated traits.

### Lineage-specific genome reduction in the *Apophysomyces*-associated *M. apophysomyceticola* clade

One of the novel species-level clusters identified by the all-versus-all ANI/dDDH analysis comprised the two *Mycetohabitans* MAGs recovered from *Apophysomyces* hosts, here provisionally designated *M. apophysomyceticola* (Fig. 1). Both *M. apophysomyceticola* MAGs were of high quality, with CheckM2 estimating 92.7% completeness and 2.1% contamination for MAG14 and 99.8% completeness with no detectable contamination for MAG15. In the whole-genome phylogeny, the *M. apophysomyceticola* MAGs formed a strongly supported clade (100% bootstrap) corresponding to the only currently sampled *Apophysomyces–Mycetohabitans* associations (Fig. 3). Consistent with the ANI results, GTDB-Tk classified both MAGs to the genus *Mycetohabitans* but did not affiliate them with any described species, flagging them as taxonomically novel based on RED scores (Fig. S4). Similarly, dDDH analysis via TYGS further supported this inference, with the MAGs not belonging to any species found in the TYGS database and being flagged as potential new species (File S5). Notably, these genomes were recovered from *Apophysomyces* hosts, whereas all previously characterized *Mycetohabitans* have been associated with *Rhizopus,* as shown in the tanglegram linking bacterial endosymbiont and fungal host phylogenies (Fig. 3). Together, these results indicate that the lineage provisionally designated *M. apophysomyceticola* comprises a distinct, previously undescribed species within *Mycetohabitans*.

In contrast to other *Mycetohabitans* spp., *M. apophysomyceticola* displayed a striking contraction of functional capacity. Functional enrichment of KEGG modules revealed the complete loss of multiple central metabolic modules (gluconeogenesis, oxidative pentose phosphate pathway), inorganic nutrient assimilation pathways (assimilatory nitrate reduction, sulphate assimilation and siroheme biosynthesis) and diverse amino acid and aromatic compound catabolic and degradation routes (lysine, proline, leucine, tyrosine, catechol and toluene/xylene) (File S12). Similarly, modules for vitamin and cofactor biosynthesis (thiamine, cobalamin and cofactor F_420_) were absent, alongside stress-response systems such as ectoine biosynthesis (File S15). Defensive and interaction-related functions were also reduced, including the loss of cell-envelope modification (dTDP-l-rhamnose biosynthesis) and antimicrobial resistance modules (vancomycin resistance d-Ala-d-Lac type, CAMP resistance MprF, beta-lactam resistance Bla system and multidrug resistance MexJK-OprM efflux pumps) (File S15). This extensive erosion of metabolic flexibility, stress tolerance and host–interaction machinery is consistent with the observed genome contraction (~2 Mb genome size of *M. apophysomyceticola* vs. ~3.5 Mb genome size of *Mycetohabitans* spp.) and suggests that *M. apophysomyceticola* is transitioning towards a more obligate symbiotic lifestyle, reliant on its *Apophysomyces* host for nutrients, co-factors and protection.

## Discussion

This study provides the most extensive survey to date of *Mycetohabitans* endosymbionts in mucoralean fungi. By screening 1,696 publicly available Mucorales SRA datasets for the presence of *Mycetohabitans* sequences, we detected *Mycetohabitans* in 46 accessions spanning 4 fungal host taxa. These included previously reported associations involving *R. microsporus*, *R. arrhizus*, *R*. homothallicus and *Apophysomyces* sp. [21,51, 98]. Positive *Mycetohabitans* SRA accessions were recovered from Brazil, the Philippines, South Africa, Germany, Japan, Pakistan, the USA and India, but detection rates varied strongly among countries and host groups. It should be noted that this variation likely reflects both biological structure and the uneven composition of public sequencing repositories, rather than true geographical prevalence alone.

However, the SRA-based results represent only one component of the known *Mycetohabitans* global distribution. Several described *Rhizopus–Mycetohabitans* associations and geographical records are absent from the screened datasets, including reports from Mexico, Australia, Georgia, Ukraine, China, Vietnam and Mozambique, alongside additional records from countries also represented in our screen [99,101]. Together, these records indicate that *Mycetohabitans* has a wider host-associated and geographical distribution than can be inferred from SRA mining alone, emphasizing both the value and the limitations of public metagenomic/genomic repositories for reconstructing endosymbiont diversity.

Genome reconstruction showed that most *Mycetohabitans*-positive datasets contained sufficient bacterial signal to recover near-complete MAGs, supporting the interpretation that these reads represent bona fide endosymbionts rather than incidental contaminants. In total, 38 *Mycetohabitans* MAGs were recovered, of which 34 were high quality and comparable in size and BUSCO completeness to available reference genomes. These MAGs substantially increased the genomic sampling of the genus and enabled genome-based species delimitation across both previously characterized isolates and endosymbionts recovered from public fungal sequencing datasets.

Previous genome-based work recognized four species-level clusters within *Mycetohabitans* [23]. By incorporating newly recovered MAGs, our all-versus-all ANI/dDDH analysis expanded this framework to nine species-level clusters [102]. These included four clusters corresponding to previously recognized species-level groups, two novel multi-isolate clusters and three novel singleton lineages represented by individual MAGs. This result indicates that species-level diversity within *Mycetohabitans* has been substantially underestimated, consistent with earlier suggestions that *Mycetohabitans* diversity is underexplored [42]. It also demonstrates that public fungal genome datasets contain hidden bacterial symbiont diversity that is not captured by culture-based or targeted endosymbiont surveys alone. Similar patterns have been reported for *Mycoavidus* and other Mucorales-associated *Burkholderiaceae*, where expanded genome sampling has revealed greater taxonomic complexity than previously recognized [97].

The expanded dataset also highlights the need for caution when assigning names to under sampled species-level lineages. For example, one MAG recovered here from a 2009 clinical isolate from the USA clustered with *M. australianus*, a species originally represented by a single Australian isolate. The original *M. australianus* BioSample does not include collection-date metadata, and because the expanded sampling now shows that this lineage is not restricted to Australia, the geographic basis or origin of this strain and name may not fully reflect the known distribution or history of the clade. To avoid introducing a similar issue for newly resolved singleton lineages, we used non-geographic provisional names; for instance, the singleton lineage represented by the only Brazilian *Mycetohabitans* MAG was provisionally designated *M. cryptica* rather than being named after its geographic origin.

The updated species delimitation also revises interpretation of previously reported host–symbiont associations. In particular, the previously broad *M. endofungorum–R. arrhizus* association was resolved into a distinct *R. arrhizus*-associated species-level lineage, here provisionally designated *M. arrhizicola*. Under this revised framework, *M. endofungorum* was recovered only from *R. microsporus* datasets, suggesting that earlier reports of *M. endofungorum* in both *R. microsporus* and *R. arrhizus* reflected the limited taxonomic resolution available before recognition of this additional species-level cluster. Our genome-resolved analyses also clarify other previously unresolved host associations. *Mycetohabitans* had recently been detected in *R. homothallicus* using 16S phylogeny, with the suggestion that it might represent a novel lineage [38]. The *R. homothallicus*-associated MAG recovered here formed a singleton species-level lineage in ANI/dDDH analyses, supporting that interpretation and provisionally designated here as *M. homothallicola*. Similarly, although *Mycetohabitans* had previously been reported from *Apophysomyces*, the symbiont had not been resolved beyond genus level [42]. The two *Apophysomyces*-associated MAGs recovered here formed a distinct species-level cluster, provisionally designated *M. apophysomyceticola*. Thus, rather than simply expanding the known host range of *Mycetohabitans*, these results refine it by showing that previously recognized host associations include distinct, genome-resolved bacterial lineages that were not captured by earlier genus-level or marker-based approaches.

Host–symbiont associations were non-random under the updated species framework. The fungal species-by-bacterial species contingency analysis showed significant departure from independence, and ParaFit detected significant global congruence between fungal and bacterial phylogenies. However, individual link tests indicated that this structure was not uniform across all associations. The two *Apophysomyces–M. apophysomyceticola* associations were the only links that remained significant after FDR correction (*q*=0.0038). Many other links were nominally significant before correction, but their adjusted *q*-values rose to 0.0755 and therefore fell just above the significance threshold. These links may represent suggestive but not statistically supported host–symbiont associations, whereas several others showed no support or were discordant. This pattern suggests that some *Mycetohabitans* lineages may show strong host association, while others may have a more complex history involving host switching, incomplete lineage sorting or under sampling of intermediate host–symbiont combinations. For example, *M. rhizoxinica* was predominantly associated with *R. microsporus* (14/16 associations) but was also detected in *R. arrhizus* (2/16), while *M. arrhizicola* showed the reciprocal pattern, occurring mainly in *R. arrhizus* (8/9) but also in *R. microsporus* (1/9). These cross-host occurrences suggest that host association is not strictly fixed for all *Mycetohabitans* spp. and may reflect occasional host switching or unresolved historical associations.

A major challenge for interpreting these associations is the frequency of fungal host misidentification. Among endosymbiont-positive datasets which all underwent host re-identification, 20.5% were misidentified or misannotated at either the species or genus level (Table 1). This finding highlights the importance of sequence-based verification when researching the Mucorales and utilizing public databases. Reliance on collection metadata alone risks propagating erroneous host–symbiont links, particularly in Mucorales, where species boundaries can be difficult to resolve using morphology or limited marker data. Because many Mucorales are also clinically relevant, improved genome-based identification is important not only for ecological and evolutionary studies but also for studies of pathogenesis and antifungal response.

Clinical datasets revealed an additional pattern; *Mycetohabitans*-positive human-associated samples were almost exclusively linked to *R. microsporus*, despite similar overall prevalence of *Mycetohabitans* in *R. microsporus* and *R. arrhizus* across all environmental and clinical datasets. No *Mycetohabitans* sequences were detected in clinical *R. arrhizus* samples, even though *R. arrhizus* environmental isolates frequently harboured *M. arrhizicola* and occasionally other *Mycetohabitans* spp. This could indicate ecological or clinical differences in the distribution of infected isolates, although the interpretation should remain cautious because public datasets are not systematically sampled and some endosymbiont-negative hosts may be misidentified. Targeted clinical sampling will be required to determine whether *Mycetohabitans* infection is genuinely enriched in clinical *R. microsporus* relative to clinical *R. arrhizus*.

Pangenome analysis revealed that expanded genomic sampling captures substantial functional diversity within *Mycetohabitans*. The *Mycetohabitans* MAG-based pangenome remained open, indicating that additional sampling is likely to reveal further gene content diversity and reinforcing that current estimates represent only part of the functional repertoire of the genus.

Functional enrichment analyses reveal a mosaic distribution of functional traits across multiple species-level lineages. Functional losses in the *M. endofungorum*-related lineages included catabolic pathways, nitrogen and respiratory metabolism, cofactor biosynthesis, secretion and cell-envelope-associated systems, metal resistance, antimicrobial resistance-associated functions and mobile element-associated genes. Because several lineages (*M. endofungorum*, *M. arrhizicola* and *M. euroasiaticus*) were represented by few genomes, these losses should be interpreted as lineage-associated hypotheses rather than definitive species traits. However, our data are consistent with an analysis that *M. endofungorum*-related isolates are enriched for toxin production, previously raised as a potential risk in the use of *R. arrhizus* in food fermentation processes [48]. Bringing this together with the host-association discussed above, the dynamic association of *M. endofungorum*-related lineages with both *R. microsporus* and *R. arrhizus*, identified from both environmental and clinical isolation sources, suggests that they may be sharing an environmental niche with the potential for movement between hosts.

One lineage, however, differed from this broader pattern of accessory gene variation by showing evidence of pronounced genome reduction. The reduced lineage comprised two *Apophysomyces*-associated MAGSs provisionally designated *M. apophysomyceticola*. These MAGs formed a coherent species-level cluster, were placed as a distinct lineage in the whole-genome phylogeny and were not assigned to any described species by GTDB-Tk or TYGS. In addition to its phylogenomic distinctness, this lineage is marked by pronounced genome reduction, with genomes of ~2 Mb compared with ~3.5 Mb in other sampled *Mycetohabitans* spp., together with extensive loss of functional modules. Enrichment analysis highlighted the absence of central metabolic routes (gluconeogenesis, oxidative pentose phosphate pathway), inorganic nitrogen and sulphur assimilation (nitrate and sulphate reduction, siroheme biosynthesis) and multiple vitamin and cofactor pathways (thiamine, cobalamin and F_420_). Likewise, the species has shed broad catabolic flexibility (amino acid degradation and aromatic compound breakdown), osmoprotection (ectoine biosynthesis) and several cell-envelope and defence systems, including dTDP-l-rhamnose biosynthesis, CAMP resistance, multidrug efflux, beta-lactam resistance and vancomycin-type resistance. The absence of these pathways is consistent with a substantial reduction in metabolic flexibility and stress tolerance. Such erosion of metabolic, defensive and interaction-associated capacity parallels patterns reported from other fungal and insect symbionts undergoing reductive evolution toward greater host dependence [50,103, 104]. Our repeated recovery of this reduced lineage from independent public *Apophysomyces* datasets suggests that this is not an isolated occurrence but instead reflects a stable host association and divergence from *Rhizopus*-associated *Mycetohabitans* spp.

The apparent loss of F420-related metabolism is particularly notable because, in *R. microsporus* symbioses, 3PG-F420 is required for establishment of a functional interaction but not for bacterial colonization per se [105]. This raises the possibility that the *Apophysomyces*-associated lineage has undergone not only extensive metabolic reduction but also lineage-specific remodelling of the molecular mechanisms used to establish and maintain endosymbiosis. More broadly, the combination of expanded species-level diversity across *Mycetohabitans* and extreme functional reduction in the *Apophysomyces*-associated clade provides a useful comparative framework for understanding how endofungal bacteria diversify across host lineages while, in some cases, evolving toward increasingly specialized and host-dependent lifestyles [106,109].

In conclusion, this work illustrates the power of mining large-scale public sequencing datasets for hidden symbiotic associations. By integrating endosymbiont detection, fungal host verification, genome reconstruction, genome-based species delimitation and MAG-focused pangenomics, we provide a new framework for understanding the diversity, distribution and functional differentiation of *Mycetohabitans* symbionts. Our results show that these associations are more widespread and taxonomically diverse than previously recognized and that some lineages, such as the *Apophysomyces*-associated clade, have undergone genome reduction and transition to obligacy.

At the same time, these findings should be interpreted within the limits of public repository data. Although reference genomes from cultured isolates were used to anchor species assignments and phylogenomic placement, the pangenome analysis focused on MAGs recovered in this study and should therefore not be considered a complete genus-wide pangenome. Key challenges also remain, including persistent host misidentification, uneven geographic sampling and the fact that public repository data are shaped by prior research priorities rather than systematic ecological sampling. As a result, some hosts, regions and sample types are likely overrepresented, whereas others remain undersampled or absent entirely. Consequently, the patterns we observe in host range, geographic distribution and apparent prevalence should be interpreted as signals within the currently available sequencing record rather than unbiased estimates of natural occurrence. Moreover, the lack of detailed metadata in most public sequencing datasets means that critical aspects of sample processing, DNA extraction methods and library preparation remain unknown. Such methodological variation may influence the recovery of both fungal and bacterial DNA and reads, potentially biassing prevalence estimates and hindering reproducibility. Future work combining environmental surveys, targeted clinical sampling and experimental validation will be essential to resolve the ecological roles, transmission dynamics and clinical implications of Mucorales*–Mycetohabitans* symbioses.

Pairwise genomic similarity among all analysed *Mycetohabitans* and *Burkholderia sensu lato* genomes and MAGs, shown as a combined triangular heatmap. The upper-right matrix displays ANI values, whereas the lower-left matrix displays dDDH values. Genomes are arranged by genomic relatedness, revealing nine species-level clusters based on accepted cut-offs of >95% ANI and >70% dDDH; species-level clusters are indicated with dotted lines. Proposed species-level lineages are labelled as *M. apophysomyceticola*, *M. arrhizicola*, *M. homothallicola*, *M. cryptica* and *M. discreta*. Labels include strain names or MAG identifiers, with source SRA accessions provided for MAG-derived assemblies.

## Supplementary material

10.1099/mgen.0.001746 Supplementary Material 1.

## Abbreviations

ANI: average nucleotide identity
BRE: *Burkholderia*-related endobacteria
dDDH: digital DNA–DNA hybridization
FDR: false discovery rate
ITS: internal transcribed spacer
MAG: metagenome-assembled genome
SRA: Sequence Read Archive
TYGS: Type (Strain) Genome Server
WGS: whole genome sequencing
