# Supplementary material for: Phylogenetically diverse Mucorales–Mycetohabitans endosymbiotic interactions identified from whole-genome sequencing using a targeted metagenomic assembly pipeline
Source: Microb Genom. 2026 Jul 14;12(7):001746. doi: 10.1099/mgen.0.001746 (PMC13367373; doi:10.1099/mgen.0.001746)
Supplement: Supplementary Material 1. [file mgen-12-01746-s001.pdf]

Figure S1 – Overview of the endosymbiont discovery and genome-recovery pipeline.
Schematic summary of the bioinformatic workflow used to identify and characterise putative Mycetohabitans-associated endosymbionts from public sequencing data. A total of 1,696 public SRA datasets were screened. Reads were downloaded, quality checked, trimmed and taxonomically classified using Kraken2 and MetaPhlAn. Datasets containing more than 1,000 Mycetohabitans-classified reads were retained for metagenomic assembly, whereas datasets below this threshold were excluded from downstream genome recovery. Assembled contigs were taxonomically classified using BLAST and Kraken2, followed by genome binning to recover putative endosymbiont and fungal host genome bins. Endosymbiont genome bins were assessed using QUAST and BUSCO prior to phylogenomic, pangenome and functional analyses. Fungal host genome bins were similarly quality assessed, followed by ITS extraction and host phylogenetic analysis.

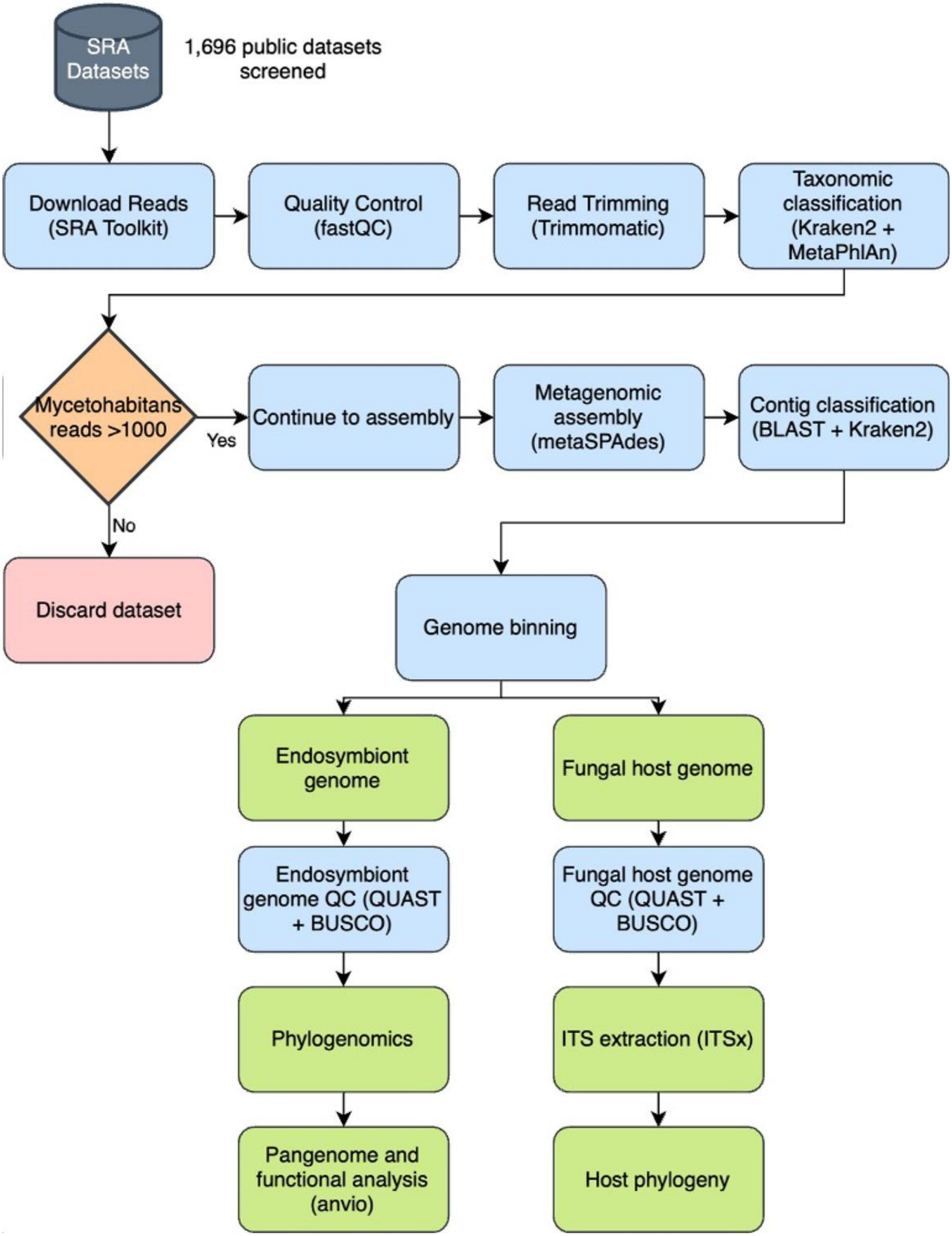
SRA Datasets
1,696 public datasets screened
Download Reads (SRA Toolkit)
Quality Control (fastQC)
Read Trimming (Trimmomatic)
Taxonomic classification (Kraken2 + MetaPhlAn)
Mycetohabitans reads >1000
Yes
No
Continue to assembly
Metagenomic assembly (metaSPAdes)
Contig classification (BLAST + Kraken2)
Discard dataset
Genome binning
Endosymbiont genome
Fungal host genome
Endosymbiont genome QC (QUAST + BUSCO)
Fungal host genome QC (QUAST + BUSCO)
Phylogenomics
ITS extraction (ITSx)
Pangenome and functional analysis (anvio)
Host phylogeny

Figure S2 – Prevalence of Mycetohabitans endosymbionts across mucoralean genera.
(A) Endosymbiont prevalence by fungal genus, showing only genera with at least one positive sample. Bars indicate the percentage of isolates with Mycetohabitans detected, with numbers above bars denoting positive/total isolates tested.
(B) Proportion of positive (green) and negative (grey) isolates for the ten most frequently sampled fungal genera. Bars represent the fraction of accessions with or without detectable endosymbionts; numbers within positive segments show positive/total isolates and the corresponding percentage. See File S9 for all countries and prevalences analysed in this dataset.

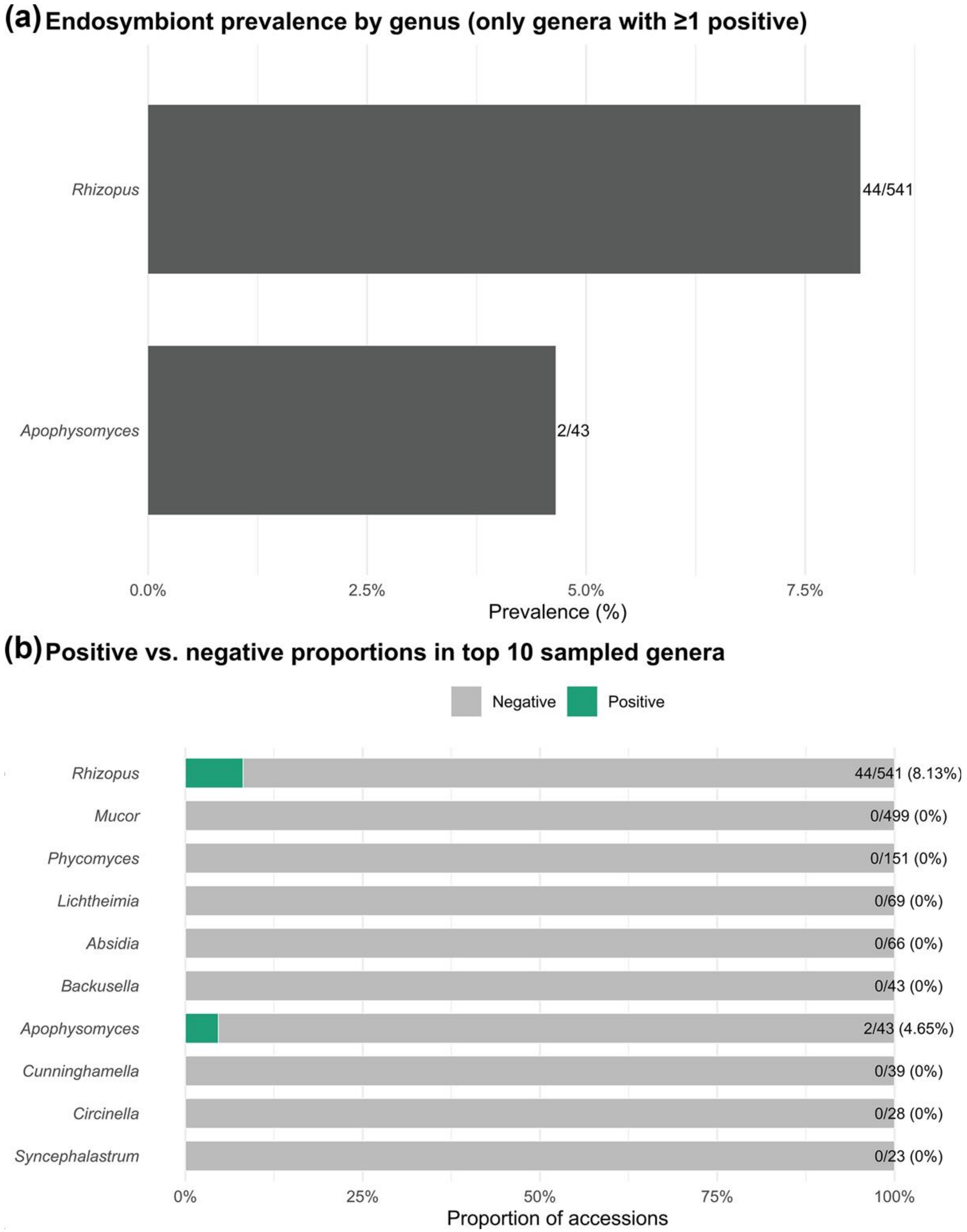
(a) Endosymbiont prevalence by genus (only genera with ≥1 positive)
Rhizopus
44/541
Apophysomyces
2/43
0.0%
2.5%
5.0%
7.5%
Prevalence (%)
(b) Positive vs. negative proportions in top 10 sampled genera
Negative
Positive
Rhizopus
44/541 (8.13%)
Mucor
0/499 (0%)
Phycomyces
0/151 (0%)
Lichtheimia
0/69 (0%)
Absidia
0/66 (0%)
Backusella
0/43 (0%)
Apophysomyces
2/43 (4.65%)
Cunninghamella
0/39 (0%)
Circinella
0/28 (0%)
Syncephalastrum
0/23 (0%)
0%
25%
50%
75%
100%
Proportion of accessions

Figure S3 – Mucorales-Mycetohabitans pairings heatmap and Chi-squared test of independence.
Heatmap showing the number of observed pairings between fungal host species and Mycetohabitans species-level lineages recovered in this study. Rows represent fungal hosts and columns represent bacterial endosymbiont species; cell values indicate the number of accessions assigned to each host–symbiont combination, with darker shading corresponding to higher occurrence. Blank cells indicate host–symbiont combinations not detected in the analysed dataset. Hierarchical clustering groups fungal hosts and bacterial lineages according to similarity in their pairing profiles. A chi-square test of independence indicated that host–symbiont pairings were non-random across the dataset ($p = 7.48 \times 10^{-11}$), supporting structured host association among Mycetohabitans lineages.

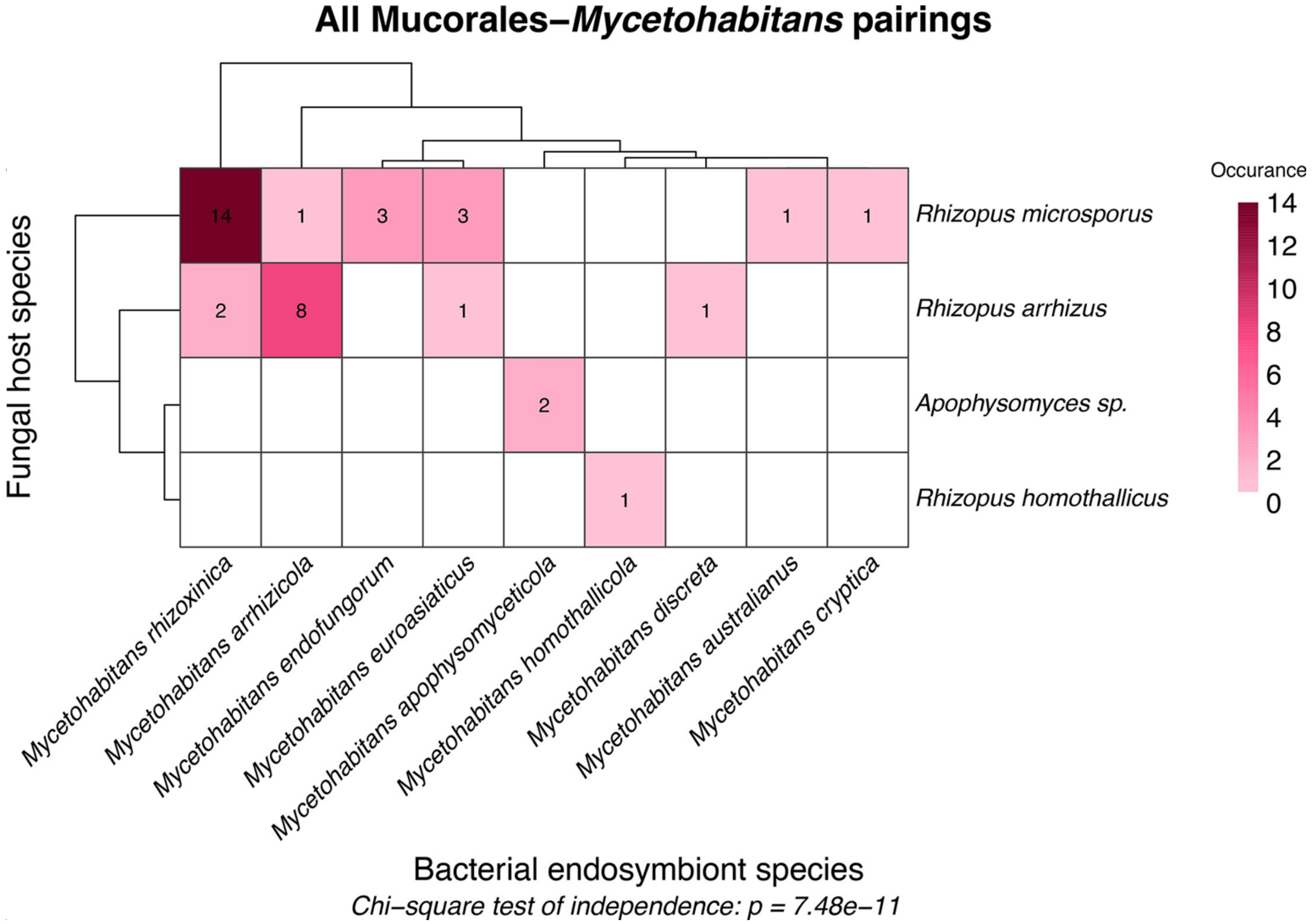

Figure S4 – Tree inferred with FastME from GBDP distances calculated from genome sequences of the Apophysomyces-associated MAGs and closely related species. The branch lengths are scaled in terms of GBDP distance formula d5. The numbers above branches are GBDP pseudo-bootstrap support values > 60 % from 100 replications, with an average branch support of 86.4 %. The tree was rooted at the midpoint.

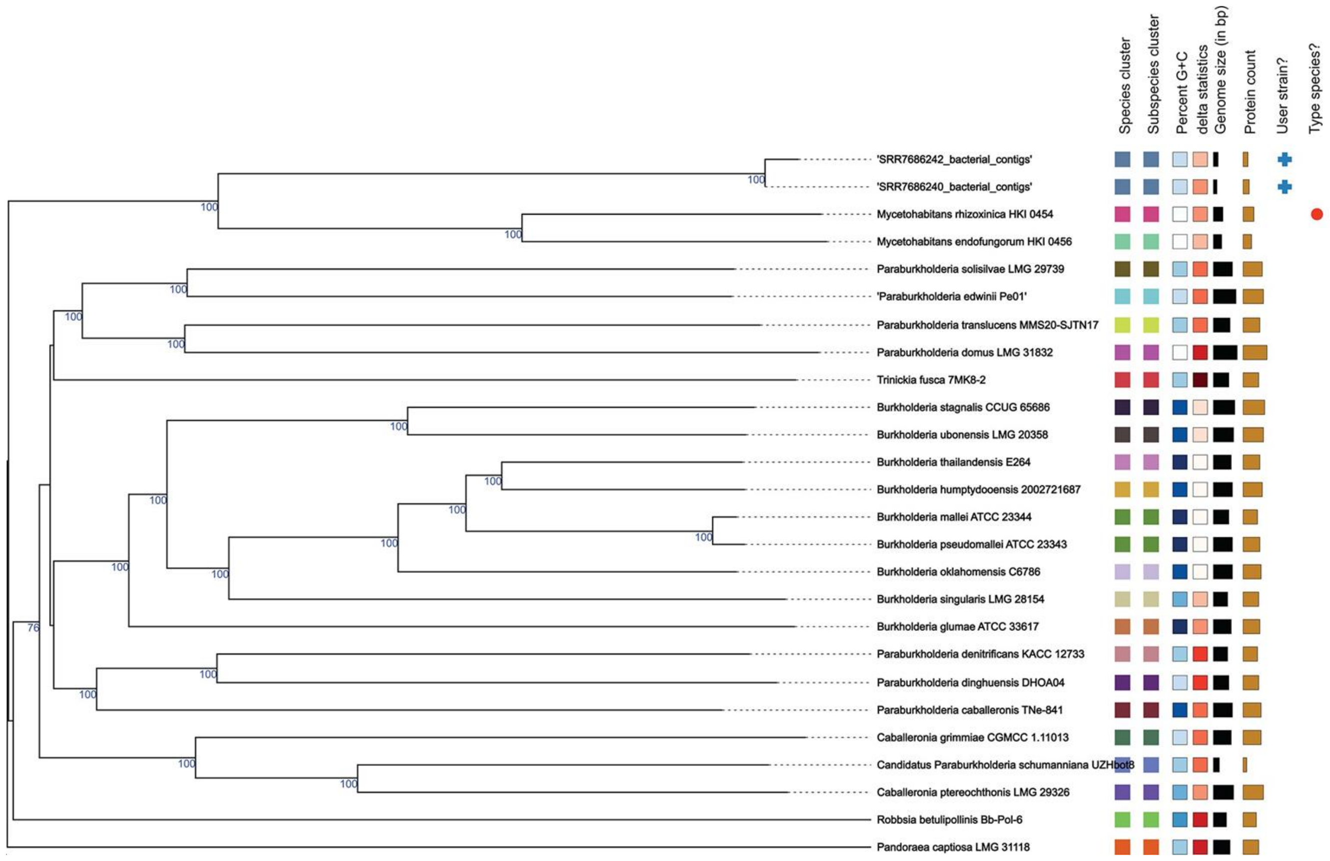

Species cluster
Subspecies cluster
Percent G+C
delta statistics
Genome size (in bp)
Protein count
User strain?
Type species?
'SRR7686242_bacterial_contigs'
'SRR7686240_bacterial_contigs'
Mycetohabitans rhizoxinica HKI 0454
Mycetohabitans endofungorum HKI 0456
Paraburkholderia solisilvae LMG 29739
'Paraburkholderia edwinii Pe01'
Paraburkholderia translucens MMS20-SJTN17
Paraburkholderia domus LMG 31832
Trinickia fusca 7MK8-2
Burkholderia stagnalis CCUG 65686
Burkholderia ubonensis LMG 20358
Burkholderia thailandensis E264
Burkholderia humptydooensis 2002721687
Burkholderia mallei ATCC 23344
Burkholderia pseudomallei ATCC 23343
Burkholderia oklahomensis C6786
Burkholderia singularis LMG 28154
Burkholderia glumae ATCC 33617
Paraburkholderia denitrificans KACC 12733
Paraburkholderia dinghuensis DHOA04
Paraburkholderia caballeronis TNe-841
Caballeronia grimmiae CGMCC 1.11013
Candidatus Paraburkholderia schumanniana UZHbot8
Caballeronia ptereochthonis LMG 29326
Robbsia betulipollinis Bb-Pol-6
Pandoraea captiosa LMG 31118
